# Metabolic alterations caused by HNF1β expression in ovarian clear cell carcinoma contribute to cell survival

**DOI:** 10.18632/oncotarget.4692

**Published:** 2015-07-31

**Authors:** Yasuaki Amano, Masaki Mandai, Ken Yamaguchi, Noriomi Matsumura, Budiman Kharma, Tsukasa Baba, Kaoru Abiko, Junzo Hamanishi, Yumiko Yoshioka, Ikuo Konishi

**Affiliations:** ^1^ Department of Gynecology and Obstetrics, Kyoto University Graduate School of Medicine, Kyoto, Japan; ^2^ Department of Obstetrics and Gynecology, Kinki University Faculty of Medicine, Osaka-Sayama, Japan

**Keywords:** ovarian clear cell carcinoma, HNF1β, metabolome analysis, aerobic glycolysis, ROS

## Abstract

HNF1β is expressed exclusively in ovarian clear cell carcinoma (OCCC) and not in other ovarian cancers, regarded as a hallmark of this tumor. This implies its central role in the unique character of OCCC, including resistance to chemotherapy, but its exact role and influence in cancer biology or the molecular bases of its function are largely unknown. Using comprehensive metabolome analysis of *HNF1β*_shRNA-stable cell lines, we show here that HNF1β drastically alters intracellular metabolism, especially in direction to enhance aerobic glycolysis, so called the “Warburg effect”. The consequence of the metabolic change contributed cell survival under stresses such as hypoxia and chemo-reagent, only when sufficient glucose supply was available. Augmented cell survival was based on the reduced ROS activity derived from metabolic alteration such as shift from oxidative phosphorylation to glycolysis and increased intracellular anti-oxidant, glutathione (GSH). One of the cystine transporters, rBAT is likely to play a major role in this GSH increase. These data suggest that HNF1β, possibly induced by stressful microenvironment in the endometriotic cyst, confers survival advantage to the epithelial cells, which leads to the occurrence of OCCC, a chemo-resistant phenotype of ovarian cancer.

## INTRODUCTION

Ovarian clear cell carcinoma (OCCC) is a relatively rare subtype among the ovarian cancers, but is a clinically important entity because it is generally resistant to chemotherapy and is known to have a worse prognosis compared with other ovarian cancers [1–4]. Another clinical feature of OCCC is its frequent occurrence in endometriotic cysts, so called “chocolate cysts”. Studies have indicated that approximately 1% of endometriotic cysts give rise to ovarian cancer [5–9], and, unlike common ovarian cancers, which are most frequently serous carcinomas, cancers occurring from endometriotic cysts are primarily clear cell and endometrioid carcinomas [8–10]. However, the reason for this remains unclear.

We have shown previously that the microenvironment within the “chocolate cyst,” which contains high levels of free iron and consequently reactive oxygen species (ROS), is extraordinarily stressful to epithelial cells within the cyst [11]. ROS are one of the major causes of cancer development, and at the same time, cancer cells must cope with various external and internal ROS for survival [12]. In addition to external ROS, an increased requirement for energy accompanied by proliferative tumor growth produces high levels of internal ROS through the mitochondrial respiratory chain [13, 14]. To avoid cell damage from these external and internal ROS, cells possess scavenger systems to eliminate ROS and minimize damage [15–18]. Indeed, the choice between cell survival and death depends on the balance between ROS production and potential for elimination.

We have also shown that OCCC has a unique gene expression profile consisting of many stress-related genes. Specifically, we suggested that the transcription factor HNF1β (hepatocyte nuclear factor 1 homeobox B), a gene specifically expressed in OCCC, distinguishes this phenotype from other ovarian cancers [19, 20]. HNF1β is a transcription factor which shares strong homology with HNF1α. Functionally, it is associated with developmental process of liver, pancreas and kidney. Clinically, heterozygous germline mutations in HNF1β is responsible for a familial forms of type 2 diabetes designated as maturity-onset diabetes of the young, subtype 5 (MODY5), which is often associated with congenital abnormalities such as polycystic kidney, pancreatic hypoplasia and genital tract abnormality [21].

In addition, overexpression of HNF1β has been reported in multiple malignancies including OCCC. So far, reported function of HNF1β includes epithelial morphogenesis and differentiation [22], regulation of urate transporter and organic anion transporters [23]. Kornfeld et. al. showed that HNF1β knockdown in mouse liver cells caused gluconeogenesis [24], suggesting that HNF1β has strong relation with glucose metabolism. However, precise mechanism how HNF1β contributes cancer biology remains to be elucidated.

We have previously demonstrated that HNF1β activates glycolysis and increases lactate production in OCCC cell line, RMG2 [25]. Regarding the unique glucose metabolism in cancer, Otto Warburg reported in 1956 that cancer cells prefer to metabolize glucose by glycolysis even in the presence of adequate oxygen [26]. This seemingly paradoxical phenomenon is called the Warburg effect and is now known as a metabolic hallmark of cancer cells, but its causal relationship with cancer progression remains largely unknown [12, 27–29]. One compelling explanation of the Warburg effect is that cancer cells gain survival advantage, such as ROS resistance and consequent apoptosis inhibition [12, 16, 27, 28], supply of biosynthetic substrates [12, 15, 16, 28], and oxygen-independent energy production [12, 15, 16, 28].

In this study, we hypothesized that HNF1β, which is expressed exclusively in OCCC confer anti-ROS activity by altering glucose metabolism, thereby contributing to carcinogenesis in high ROS environment of endometriotic cysts and cell survival in OCCC. We attempted to explore the full extent of the metabolic alterations caused by HNF1β in OCCC using a comprehensive metabolic assay. Additionally, we investigated the biological influences of these alterations.

## RESULTS

### OCCC had a unique metabolic feature compared with other histological subtypes of EOC

To identify characteristics specific for the OCCC subtype, we first performed gene set enrichment analysis (GSEA: http://www.broadinstitute.org/gsea/index.jsp) using the microarray datasets GSE39204, which contain 64 ovarian cancer clinical samples from Kyoto University Hospital, and GSE6008, which contain 99 ovarian cancer clinical samples [30]. The samples in each dataset were divided into the OCCC group and the non-OCCC group, and gene sets enriched in the OCCC group were extracted and analyzed.

**Figure 1:**
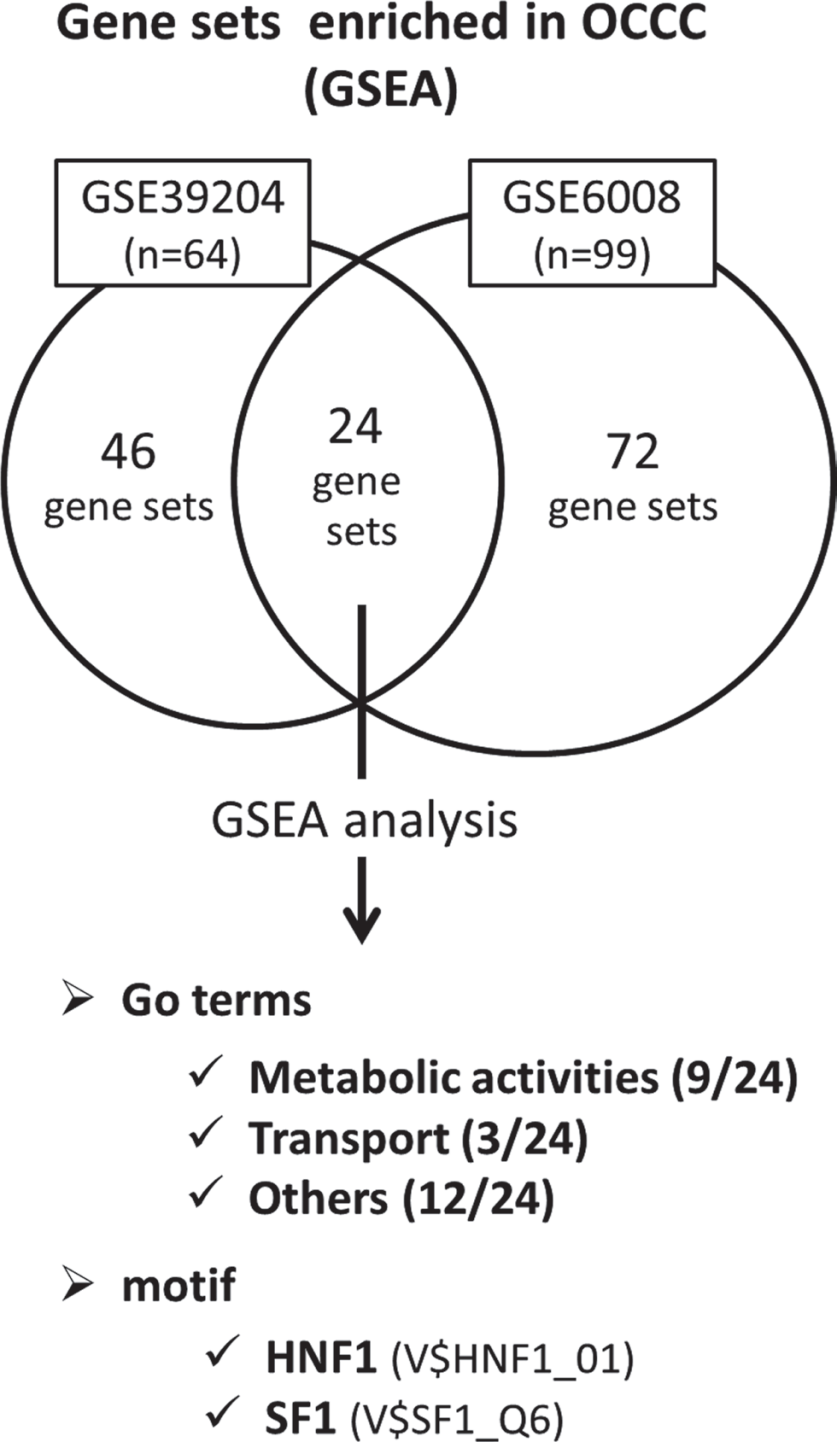
Gene expressions enriched in OCCC detected by GSEA analysis between OCCC and non-OCCC The microarray datasets GSE39204 and GSE6008 were divided into the OCCC group and the non-OCCC group, and analyzed using GSEA. In GSE39204, 46 gene sets were enriched in the OCCC group. In GSE6008, 72 gene sets were enriched in the OCCC group. 24 gene sets were enriched in both GSE39204 and GSE6008, in which 9 were metabolic activity-related. In addition, 2 transcription motifs of HNF1 and SF1 were enriched in the OCCC group in both GSE39204 and GSE6008. Detailed data including individual gene sets are shown in Supplementary Table 1a – 1c.

In GSE39204, 46 gene sets enriched in the OCCC group contained 15 metabolic activity-related gene sets. In GSE6008, 72 gene sets enriched in the OCCC group contained 13 metabolic activity-related gene sets. Among the 24 gene sets enriched both in GSE39204 and GSE6008, 9 metabolic activity-related gene sets were present. The ratio of metabolic activity-related gene sets in these groups was significantly higher than the ratio in the total gene sets (Msig DB C5_all v4.0) (GSE39204: *p* < 0.0001, GSE6008: *p* = 0.0223, both: *p* < 0.0001) (Fig. 1, detailed data are shown in Table S1a and S1b).

In addition, GSEA revealed that 2 transcription motifs of HNF1 (hepatocyte nuclear factor 1) and SF1 (splicing factor 1) were enriched in the OCCC group in both GSE39204 and GSE6008 (Msig DB C3.tft.v4.0) (Table S1c), which suggested that these transcription factors may be associated with the unique metabolic features of OCCC (Fig. 1).

### HNF1β drastically changed intracellular glucose metabolites

**Figure 2:**
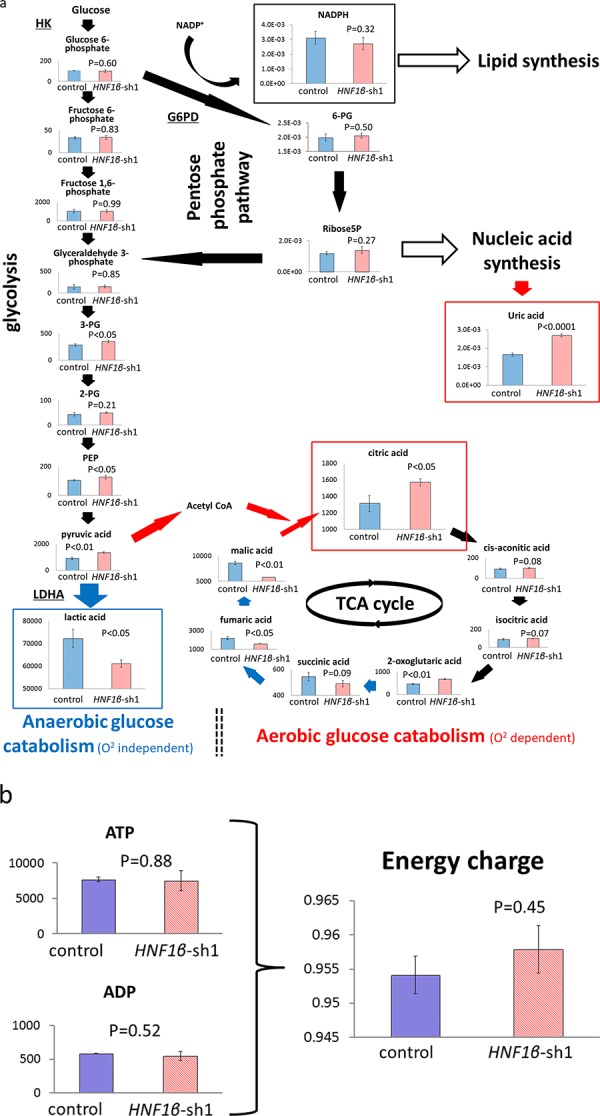
Comparison of intracellular metabolism between HNF1β-high control cells and HNF1β knockdown cells by comprehensive metabolic analysis Control: RMG2 control cells, *HNF1β*_sh1 : RMG2 *HNF1β*_sh1 cells. The amount of metabolites used was pmol /10^6^ cells. *n* = 3. **a.** Comparison of glucose metabolism including glycolysis and oxidative phosphorylation (TCA cycle) between RMG2 control cells and *HNF1β*_sh1 cells. **b.** Comparison of energy charge between RMG2 control cells and *HNF1β*_sh1 cells.

To explore the influence of HNF1β on metabolic activity in OCCC, we performed comprehensive *in vitro* analysis of intracellular metabolism in HNF1β knockdown RMG2 cells (*HNF1β*_sh1) and control cells (control) that were established as described [25]. A total of 193 peaks were identified as candidate metabolites by this assay, of which 88 metabolites were significantly altered in *HNF1β*_sh1 cells. Of these, 52 metabolites were decreased by at least 0.7-fold and 20 were increased by at least 1.4-fold (Table S2a & S2b). Specifically, lactic acid, the final product of anaerobic glycolysis, was significantly decreased in *HNF1β*_sh1 cells. In contrast, citric acid, the first metabolite of the TCA cycle following integration of acetyl CoA into this cycle from the glycolytic process, was significantly increased in *HNF1β*_sh1 cells (*p* < 0.05 for both), and malic acid that was to be converted to citric acid if pyruvic acid was provided to the TCA cycle via acetyl CoA, was decreased, suggesting that oxidative phosphorylation is more active in these cells (or less active in HNF1β-high cells by acetyl CoA supply stagnation) (Fig. 2a). Regarding as lactic acid decrease in *HNF1β*_sh1 cells, there are other mechanisms that could control the lactic acid level such as lactic acid transporters and another lactic acid synthesis pathway. However, in microarray analysis, the expression of *SLC16A1* that encodes lactic acid import transporter MCT1 was high, and that of *SLC16A3* that encodes lactic acid export transporter MCT4 was low in *HNF1β*_sh1 cells and in non-clear cell samples (Fig. S1), suggesting that lactic acid transporters expressed not to decrease lactic acid, but to compensate it. With respect to the pathway for lactic acid synthesis from pyruvate via glucose-alanine cycle, in spite of low level of lactic acid, both the alanine and pyruvate showed high level in *HNF1β*_sh1 cells in the metabolome analysis (Fig. S2). These results collectively suggested that lactic acid accumulation in HNF1β-high control cells is derived from increased glycolysis, but not from other mechanisms. Conclusively, it is suggested that HNF1β causes a metabolic shift from the TCA cycle to glycolysis. In contrast, regarding the pentose-phosphate-pathway (PPP), which is generally activated by aerobic glycolysis and thought to supply lipid and nucleotide substrates, neither 6 phosphogluconic acid (6 PG), the first metabolite of PPP, nor NADPH, the key substrate of lipid synthesis, were significantly changed (Fig. 2a). As for nucleotide synthesis, uric acid, the last product of nucleotide metabolism, increased significantly in *HNF1β*_sh1 cells (*p* < 0.0001) (Fig. 2a), which might be the result of significant G6PD increase. These data indicated that HNF1β does not activate PPP even during aerobic glycolysis. Likewise, regarding as the energy supply that is thought to be supported by aerobic glycolysis under certain conditions, neither ATP nor energy charge levels ((ATP+1/2ADP) / (ATP+ADP+AMP)) were significantly changed (Fig. 2b).

We also assessed the expression levels of the *HK1*, *G6PD*, and *LDHA* genes, which are assumed to be related to aerobic glycolysis. In *HNF1β*_sh1 cells, the expression of *HK1* and *LDHA* was significantly decreased (*p* < 0.0001), but the expression of *G6PD* was significantly increased (*p* < 0.0001) (Fig. S3a). In addition, based on analysis of the clinical dataset GSE39204, *HK1* and *LDHA* were significantly higher in the OCCC group than in the non-OCCC group, and they were significantly correlated with the expression of *HNF1β* (*p* < 0.0001). There was no significant difference in the expression of *G6PD* between the two groups (Fig. S3b).

### HNF1β expression allowed OCCC cells to survive in hypoxic conditions

**Figure 3:**
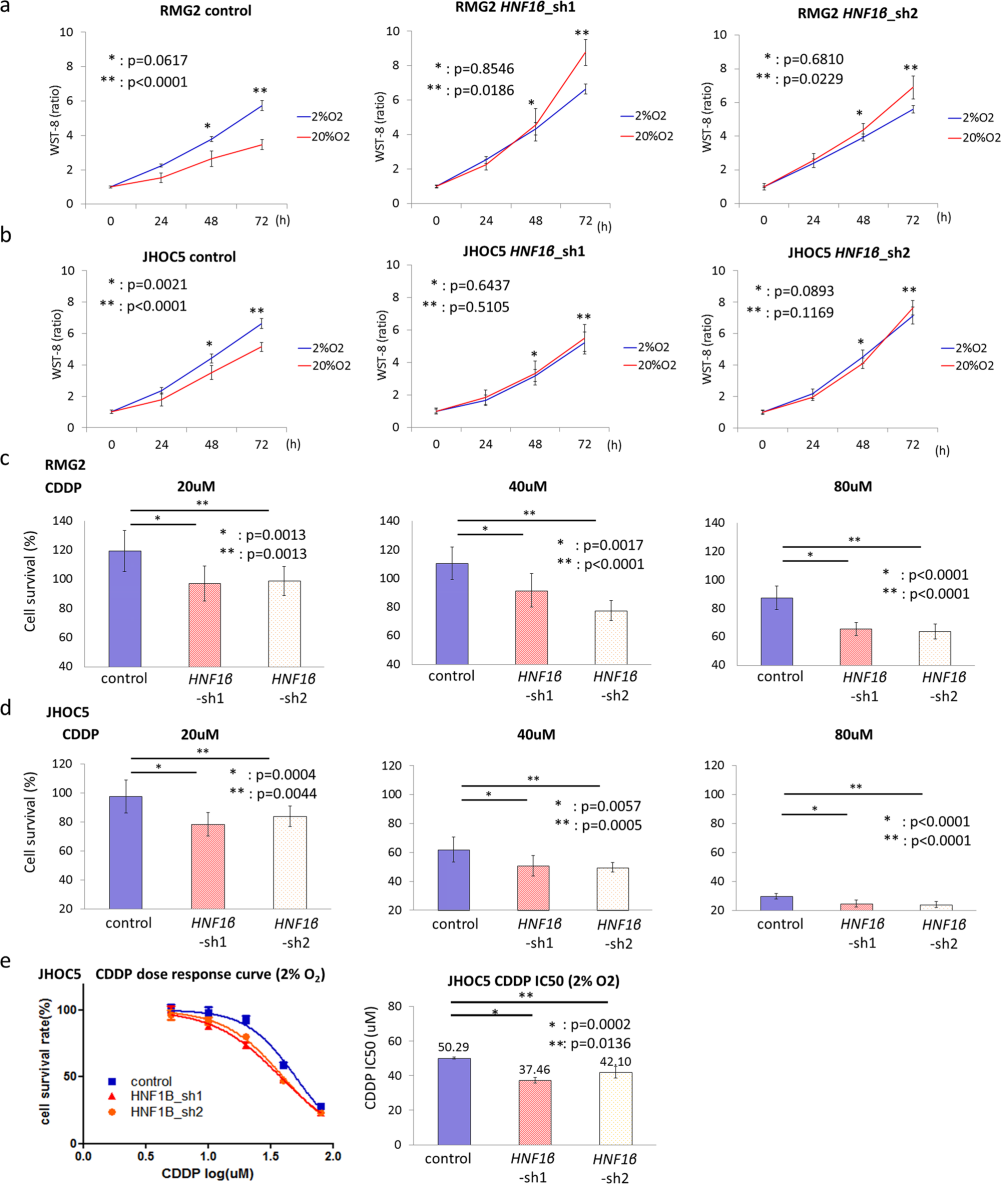
HNF1β knockdown impairs adaptation of OCCC cells to hypoxic conditions **a, b.** Comparison of cell survival in hypoxia among control, *HNF1β*_sh1, and *HNF1β*_sh2 cells. (a) RMG2 and (b) JHOC5 cells were cultured in both 20% O_2_ and 2% O_2_ and number of live cells was measured by the WST-8 method. *n* = 10. **c, d.** CDDP resistance in 2% O_2_ conditions. Control, *HNF1β*_sh1, and *HNF1β*_sh2 cells were exposed to 0, 20, 40, or 80 μM CDDP for 24 hours. Cell survival was calculated as the WST-8 value of CDDP 20, 40, or 80 μM divided by that in the absence of CDDP (0 μM CDDP) in (c) RMG2 and (d) JHOC5 cells. *n* = 10. **e.** CDDP dose response curve and IC50 of JHOC5 cell lines.

We performed functional assays using 2 types of HNF1β knockdown lines (*HNF1β*_sh1, sh2) of both RMG2 and JHOC5 OCCC cells (Fig. S4a & S4b). To explore whether HNF1β expression is associated with cell adaptation to hypoxia, we performed WST assays in both 2% and 20% O_2_. In RMG2 cell lines, the number of live HNF1β knockdown cells significantly decreased in 2% O_2_ compared to those in 20% O_2._ In contrast, in both RMG2 and JHOC5 cell lines, HNF1β-high control cells showed increased number of live cells in 2% O_2_ (Fig. 3a & 3b). We further assessed the chemo-resistance of RMG2 and JHOC5 cells under hypoxic conditions. Cell survival, which was assessed by the ratio of the number of cells alive after 24 hours exposure to 20, 40, or 80 μM CDDP to the number of cells alive after 24 hours without CDDP, was significantly decreased in HNF1β knockdown cells (*p* < 0.01 for both cell types) (Fig. 3c & 3d). Furthermore, JHOC5 cells, under hypoxic condition, showed significantly low IC50 of CDDP in HNF1β knockdown cells (*p* < 0.02) (Fig. 3e).

### HNF1β-induced cell survival was abrogated under glucose deprivation

**Figure 4:**
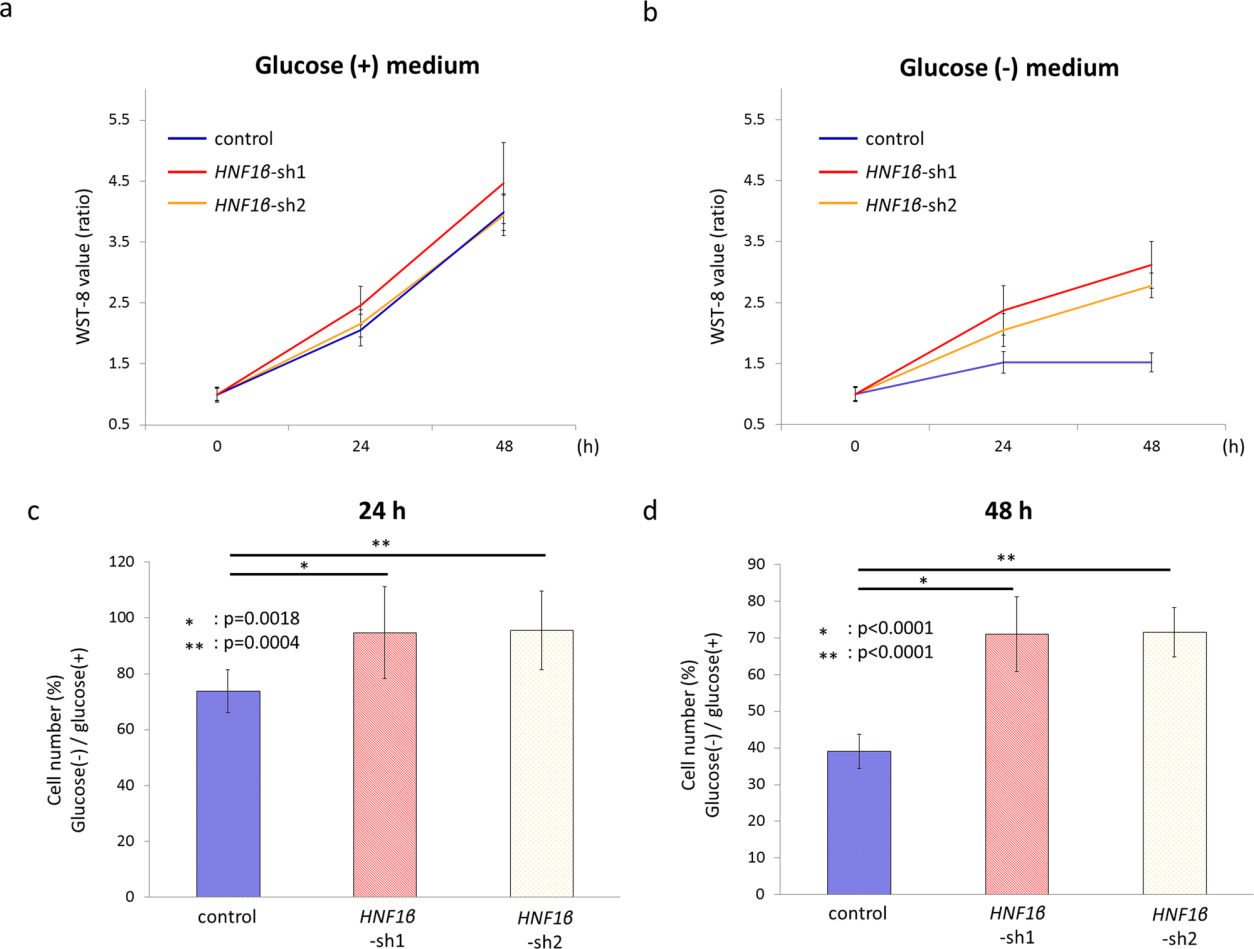
HNF1β knockdown rescued the cell survival under glucose deprivation **a, b.** Control, *HNF1β*_sh1, and *HNF1β*_sh2 RMG2 cells were cultured (a) in normal growth medium (glucose (+) medium) and (b) in glucose-deprivation medium (glucose (−) medium) in normoxic condition, and cell numbers were measured every 24 hours by WST-8 assay. The WST-8 value rate was calculated as the WST-8 value in 0, 24, or 48 hours divided by that in 0 h. **c, d.** Cell number percentages of glucose (−) / glucose (+) medium were calculated using the WST-8 values of each cells (c) in 24 hours culture and (d) in 48 hours culture respectively.

Control, *HNF1β*_sh1, and *HNF1β*_sh2 RMG2 cells were cultured in normal growth medium (glucose (+) medium) and in glucose-deprivation medium (glucose (−) medium) in normoxic condition, and cell numbers were measured every 24 hours by WST-8 assay. In glucose (+) medium, *HNF1β*_sh1, and *HNF1β*_sh2 RMG2 cells had similar number compared with control cells (Fig. 4a). By contrast, in glucose (−) medium, the number of live cells were significantly less in control RMG2 which had high HNF1β expression (Fig. 4b, 4c, & 4d), suggesting that HNF1β-induced cell survival effect is highly dependent on the glucose supply.

### HNF1β reduced intracellular ROS and contributed to oxidative stress resistance

The oxygen independent nature of aerobic glycolysis also provides an advantage by decreasing oxidative phosphorylation in the TCA cycle, which is the main source of intracellular ROS.

**Figure 5:**
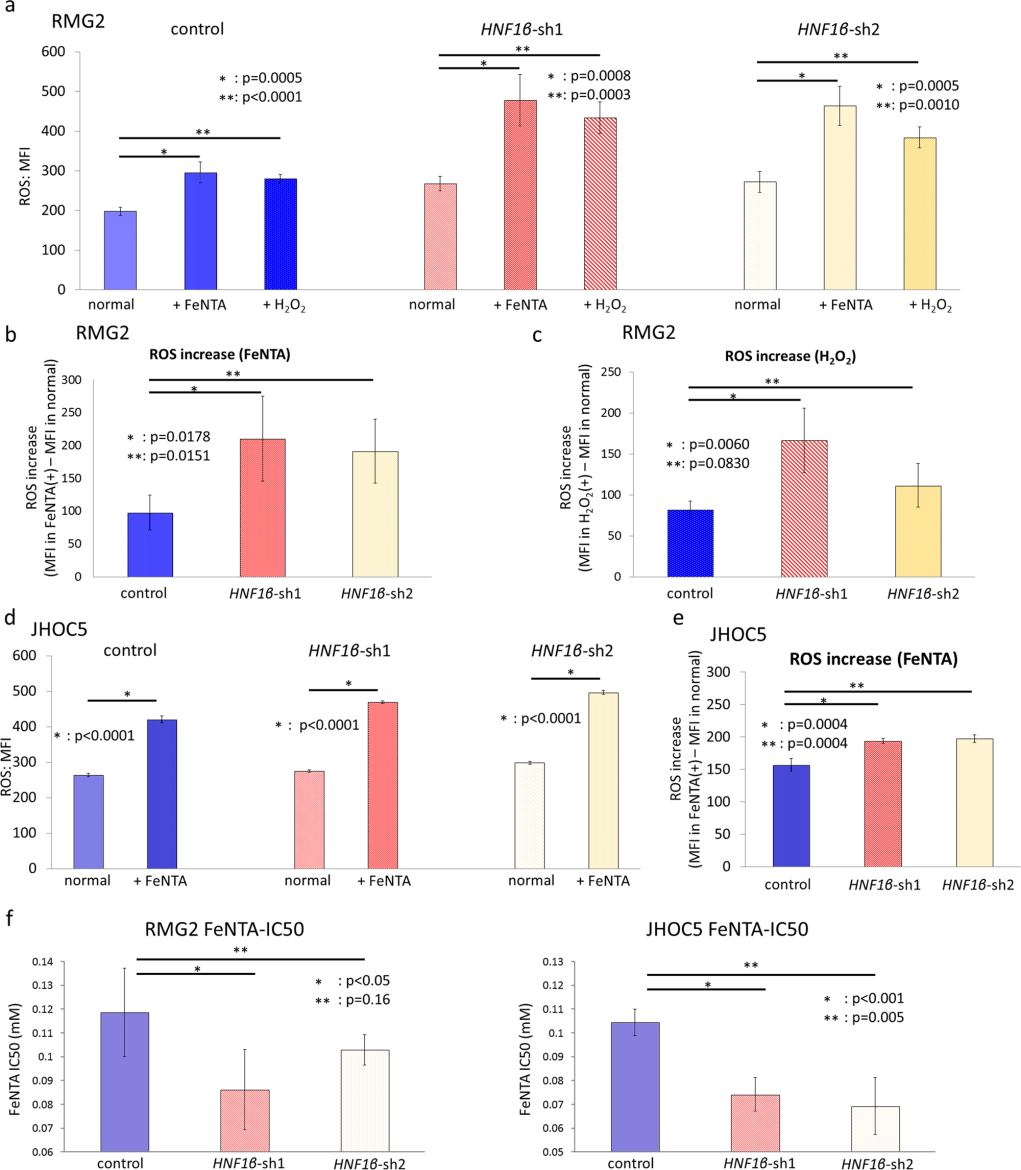
HNF1β knockdown increases intracellular ROS and decreases resistance to oxidative stress in OCCC cells The intracellular ROS activity level of control, *HNF1β*_sh1, and *HNF1β*_sh2 cells (both RMG-2 and JHOC5) was detected by CellROX^®^ Deep Red Reagent and measured by flow cytometry. **a.** RMG2 cell lines were cultured in normal medium, in medium with 0.5 mM FeNTA for 2 hours, and in medium with 0.5 mM H_2_O_2_ for 1 hour. Intracellular ROS activity levels of them were assessed by MFI (mean fluorescence intensity). *n* = 6. **b.** ROS increase by FeNTA in each cell lines was determined as (MFI of intracellular ROS activity in medium with 0.5 mM FeNTA) – (MFI of intracellular ROS activity in normal medium). **c.** ROS increase of each RMG2 cells by H_2_O_2_. **d.** Intracellular ROS activity levels of JHOC5 cell lines cultured in normal medium and in medium with 0.5 mM FeNTA for 2 hours. *n* = 6. **e.** ROS increase of each JHOC5 cells by FeNTA. **f.** FeNTA IC50 value for RMG2 and JHOC5 cells.

To explore whether HNF1β expression is associated with ROS regulation and consequent resistance to oxidative stress, we performed HNF1β redox functional assays. HNF1β knockdown significantly increased intracellular ROS activity levels in both RMG2 and JHOC5 (*p* < 0.001) (Fig. 5a & 5d). Moreover, when these cells were cultured in conditions with extracellular oxidative stress such as medium containing ferric nitrilotriacetate (FeNTA), an iron chelate, or H_2_O_2_, a well-known extra/intracellular oxidative agent, increase in intracellular ROS activity was significantly more prominent in HNF1β knockdown cells (*p* < 0.005) (Fig. 5a – 5e). This consequently decreased the IC50 of FeNTA in HNF1β knockdown cells (Fig. 5f).

### HNF1β expression was associated with intracellular GSH levels

**Figure 6:**
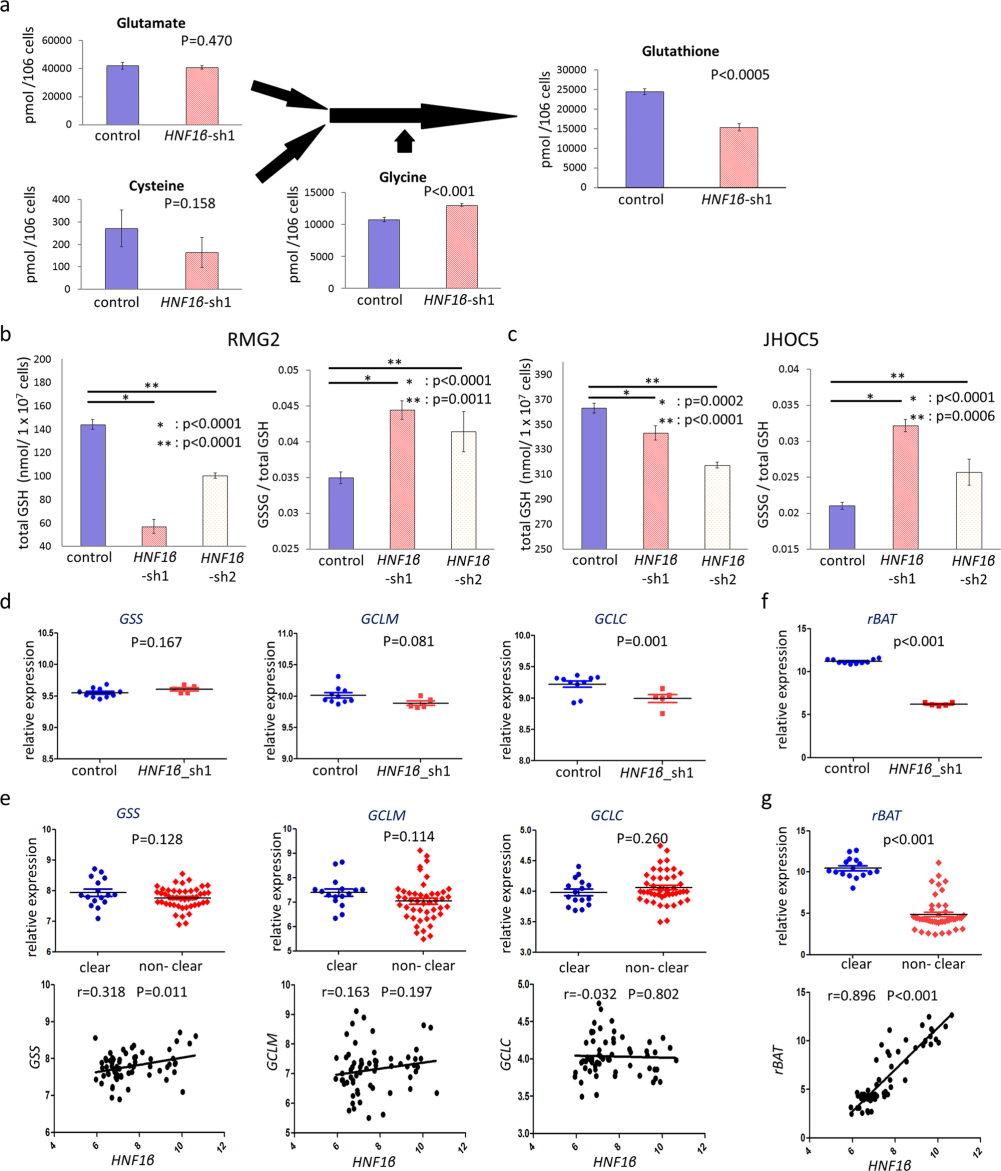
HNF1β knockdown decreases intracellular GSH levels via expression of the cystine transporter rBAT **a.** GSH synthesis pathway in the metabolome analysis. Control: RMG2 control cells, *HNF1β*_sh1 : RMG2 *HNF1β*_sh1 cells. *n* = 3. **b, c.** Intracellular GSH and the ratio of GSSG to total GSH in control, *HNF1β*_sh1, and *HNF1β*_sh2 cells of (b) RMG2 and (c) JHOC5. They were measured by the enzyme recycling method. **d, f.** Expressions of (d) GSH synthesis related genes (*GSS*, *GCLM*, and *GCLC*) and (f) a cystine transporter subunit coding gene *rBAT* were analyzed using the microarray dataset GSE37290 (RMG2 control, *HNF1β*_sh1 cell lines). **e, g.** Differences in expression levels of (e) GSH synthesis related genes and (g) *rBAT* between the OCCC group (clear) and the non-OCCC group (non-clear) were analyzed using the clinical ovarian cancer dataset GSE39204. The correlations of their expressions with that of HNF1β were additionally shown (lower columns).

To elucidate ROS regulation by HNF1β, we further evaluated the metabolism of intracellular glutathione (GSH), a primary intracellular redox regulator. Metabolome analysis in RMG2 cells showed that intracellular GSH significantly decreased in *HNF1β*_sh1 cells (*p* < 0.0005) (Fig. 6a). In GSH substrates, cysteine, which is the rate-limiting metabolite of GSH synthesis, decreased, glutamate was not altered, and glycine significantly increased in *HNF1β*_sh1 cells (*HNF1β*_sh1 /control: 0.60 (*p* = 0.158), 097 (*p* = 0.470), 1.21 (*p* < 0.001), respectively) (Fig. 6a). We confirmed the results of the metabolome analysis by another method in RMG2 and JHOC5. Using an enzyme recycling method, we assessed total intracellular GSH and the ratio of GSSG (the oxidized form of GSH) to total GSH. HNF1β knockdown significantly decreased intracellular GSH and increased the ratio of GSSG to total GSH (Fig. 6b & 6c). These results indicate that HNF1β increases intracellular GSH and, thus, enhances the redox potential of OCCC cells.

Next, we investigated the mechanism by which HNF1β increased intracellular GSH levels. Messenger RNA expression levels of GSH synthesis-related synthases, including glutamate-cysteine ligase regulatory subunit (GCLM), glutamate-cysteine ligase catalytic subunit (GCLC), and glutathione synthase (GS), as well as GSH transporters of the substrates glutamate, glycine, and cysteine, were analyzed by microarray. Among the synthase-coding genes, only *GCLC* expression was significantly decreased in the RMG2 *HNF1β_*sh1 cells (*p* < 0.05) (Fig. 6d), whereas it was not significantly associated with HNF1β expression in the clinical dataset GSE39204 (Fig. 6e). Moreover, none of the synthases displayed significantly higher expression in the OCCC group (Fig. 6e). In contrast, among the GSH substrate-related transporter-coding genes, *rBAT* / *SLC3A1*, which encoded a subunit of the b^0,+^ amino acid transporter, was significantly decreased by HNF1β knockdown (*p* < 0.001) (Fig. 6f). Furthermore, *rBAT* was significantly higher in the OCCC group and was also significantly correlated with *HNF1β* expression (*p* < 0.001) (Fig. 6g).

### HNF1β induced rBAT expression increased intracellular GSH in OCCC

**Figure 7:**
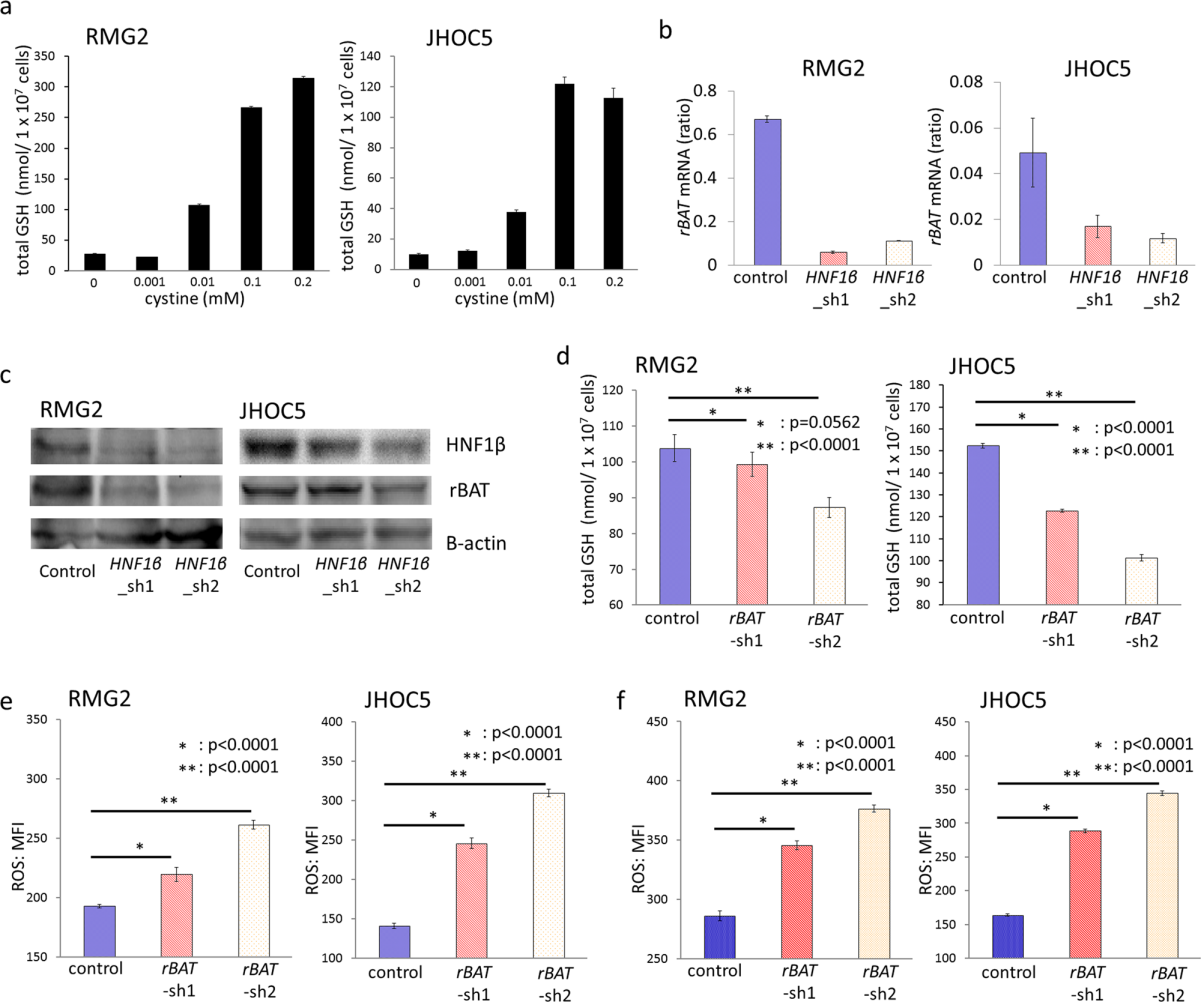
rBAT knockdown decreases intracellular GSH and increases intracellular ROS activity **a.** RMG2 and JHOC5 cells were cultured in the growing medium with 0, 0.001, 0.01, 0.1, and 0.2 mM cystine respectively, and intracellular GSH of each cells were measured using the enzyme recycling method. *n* = 6. **b.** Expression levels of *rBAT* mRNA in control, *HNF1β*_sh1, *HNF1β*_sh2 cell lines of RMG2 and JHOC5 were detected by RT-PCR. *n* = 4. **c.** Western blotting of rBAT protein in control, *HNF1β*_sh1, *HNF1β*_sh2 cell lines of RMG2 and JHOC5. **d.** Intracellular GSH in control, *rBAT*_sh1, and *rBAT*_sh2 cells of RMG2 and JHOC5, measured by the enzyme recycling method. *n* = 6. **e, f.** Intracellular ROS activity levels detected by CellROX^®^ Deep Red Reagent and measured by flow cytometry. RMG2 and JHOC5 control, *rBAT*_sh1, and *rBAT*_sh2 cells were cultured (e) in normal growth medium and (f) in medium with 0.5 mM FeNTA for 2 hours. *n* = 6.

In GSH synthesis, cysteine is known to be the rate-limiting substance because the total levels of cysteine are much lower in cells than the levels of other molecules. Most intracellular cysteine is taken into cells in its oxidized form, cystine. Therefore, we investigated whether the cystine supply altered intracellular GSH. Indeed, decreasing the cystine supply by altering the cystine concentration in the growth medium reduced intracellular GSH (Fig. 7a).

The cystine supply is generally regulated by cystine transporters in live cells. One of these transporters, *rBAT* (related to b^0,+^ amino acid transporter), was significantly decreased by HNF1β knockdown (*p* < 0.001) (Fig. 6f & 7b). Expression of rBAT protein was also decreased by HNF1β knockdown in both RMG2 and JHOC5 cells (Fig. 7c).

Based on these findings, we further investigated whether induction of rBAT by HNF1β led to GSH synthesis, using 2 types of rBAT knockdown lines (*rBAT*_sh1, sh2) of both RMG2 and JHOC5 OCCC cells (Fig. S4c & S4d). rBAT knockdown significantly reduced intracellular GSH both in RMG2 and in JHOC5 cells (Fig. 7d). Moreover, the intracellular ROS activity level was significantly increased by rBAT knockdown with or without external oxidative stress by FeNTA (*p* < 0.0001) (Fig. 7e & 7f). These results indicate that rBAT expression decreases intracellular ROS activity by promoting GSH synthesis.

## DISCUSSION

It is well known that cancer cells have unusual metabolic patterns relative to normal cells. A common example of this is the intracellular metabolic shift from oxidative phosphorylation to glycolysis, which is called the Warburg effect [27–29]. Although the Warburg effect is a universally observed phenomenon in various types of cancer cells [31–33], its causal relationship with cancer biology remains unclear. We have previously shown that HNF1β, a molecule that is expressed in high frequency and specificity in OCCC, facilitates glucose uptake and promotes concomitant glycolysis, leading to increased lactic acid production [25]. In this study, we started by confirming our previous observation [19, 20, 34] that OCCC has a characteristic gene expression profile that specifically includes enriched metabolism-related genes. Indeed, HNF1 transcription motif and metabolism-related gene sets were enriched in OCCC based on GSEA analysis using ovarian cancer data sets GSE39204 and GSE6008 (Fig. 1 & Table S1a – S1c). These data led us to investigate the broader spectrum of metabolic activity associated with HNF1β, using a comprehensive metabolite analysis. In addition, we attempted to elucidate the mechanism and biological consequence of the unique metabolism caused by HNF1β.

Comprehensive intracellular metabolites analysis revealed that HNF1β dynamically altered intracellular metabolism. First, we focused on glucose metabolism. As we have previously reported [25], lactic acid, the final product of anaerobic glycolysis, was significantly decreased in HNF1β knockdown cells (Fig. 2a). Supportively we confirmed that this lactic acid decrease was not caused by altered lactic acid transport, or another synthetic pathway. Although facilitated lactic acid accumulation by lactic acid transporter MCTs (Monocarboxylate Transporters) was reported in some cancers [35, 36], the expression of lactic acid import transporter MCT1 was low, and the lactic acid export transporter MCT4 was high in HNF1β-high cells (Fig. S1). Alanine or pyruvate, intermediates of another lactic acid synthesis pathway, also did not increase in metabolome assay in HNF1β-high cells (Fig. S2 & 2a). These results indicated that HNF1β, by glycolysis, increased lactic acid production and accumulation, which consequently caused upregulation of lactic acid export. At the same time, citric acid, the first metabolite of the TCA cycle following integration of acetyl CoA into this cycle from the glycolytic process, was significantly increased (Fig. 2a). The process converting malic acid to citric acid is impaired, leading to malic acid accumulation in HNF1β-high OCCC cells (Fig. 2a). These results suggest that HNF1β overexpression in OCCC leads to the metabolic shift from oxidative phosphorylation in the TCA cycle to glycolysis even in aerobic condition, namely, the Warburg effect.

In addition to the results of metabolome analysis, microarray data also indicated that key genes of the Warburg effect, such as *HK1*(Hexokinase-1) that supports glucose supply through phosphating glucose to glucose-6-phosphate, and *LDHA* that supports conversion pyruvate to lactic acid, [11, 37–39] were significantly higher in HNF1β-high OCCC cells (Fig. S3a). In the analysis of the clinical dataset GSE39204, *HK1* and *LDHA* were also expressed at higher levels in the OCCC group than in the non-OCCC group. Expression levels of these genes were correlated significantly to the expression of HNF1β (*p* < 0.0001) (Fig. S3b). Collectively, these results suggest that HNF1β contributes to enhance the Warburg effect. There are reports indicating that activation of specific genes, especially oncogenes such as Ras or Akt, causes similar metabolic shift [28], suggesting that genetic events associated with cancer development occasionally accompany metabolic alteration.

Several advantages of the Warburg effect have been proposed in cancer cells [12, 16, 26–28], one of which is energy support. Although anaerobic glycolysis is inefficient in energy production, it enables cells to acquire needed energy more rapidly than with oxidative phosphorylation in TCA cycle, both in aerobic and anaerobic conditions as long as abundant glucose is provided [12, 16]. Since a majority of the cancer cells grow rapidly than normal cells, they usually require excessive energy support. However, in this study, we found no evidence that HNF1β increased intracellular energy supplies at least under normoxic conditions (Fig. 2b). A second possible advantage of the Warburg effect is the supply of substrates, especially lipids and nucleotides, which are required for cellular proliferation. It is proposed that the Warburg effect activates the pentose-phosphate-pathway which increases the supply of these substrates [12, 16, 28]. However, HNF1β did not seem to activate this pathway. Neither the levels of 6 PG, the first metabolite of PPP nor NADPH, the key substrate of lipid synthesis, were significantly changed (Fig. 2a). Clinically, OCCC is known as a relatively slow-growing tumor, which suggests that OCCC particularly does not necessarily need to utilize these benefits of the Warburg effect. These data are consistent with our previous report that HNF1β, unlike oncogenes, decreases cell proliferation of OCCC in normal oxygen condition [25]. The third advantage of the Warburg effect is to support cell survival with unstable oxygen supply in cancer microenvironments [12, 16, 27, 28]. In this study, HNF1β-high OCCC cells had a survival advantage especially under hypoxic conditions (Fig. 3a & 3b). Furthermore, these cells were also more resistant to CDDP treatment not in nomoxic condition but in hypoxic conditions compared with HNF1β knockdown cells (Fig. S5a, S5b, & 3c–3e). It is notable that cell survival advantage was exerted only under abundant glucose supply. Generally, glucose dependency is thought to be one of the weak points of the Warburg effect. Other groups have reported that glucose deprivation is critical in cancers displaying the Warburg effect [37, 47, 48]. On the other hands, it has been also reported that some cancers compensate ineffective energy production of glycolysis by energy production using glutamine via glutaminolysis [49, 50]. In our metabolome assay, however, HNF1β increased neither glutamate nor α-ketoglutarate (2-OG) in the pathway of glutaminolysis (Fig. 2a), suggesting that HNF1β does not facilitate glytaminolysis. As expected, survival benefit of HNF1β-high OCCC cells was significantly impaired in glucose deprivation, suggesting that cell survival of HNF1β-high OCCC cells is highly dependent on glucose uptake and consumption (Fig. 4a – 4d).

Higher ROS activity readily leads to initiation of apoptosis. Cell survival advantage by the Warburg effect had been mostly ascribed to the detoxication of intracellular ROS. Oxidative phosphorylation in the TCA cycle is the primary source of intracellular ROS, and the Warburg effect represses it by increasing the oxygen-independent glycolysis [14, 17, 40, 41]. Therefore, we measured intracellular ROS activity in OCCC cells with high and low HNF1β expression. Our results indicated that, in normal medium, HNF1β-high OCCC cells had lower levels of intracellular ROS (Fig. 5a & 5d), suggesting that HNF1β expression is likely to reduce intracellular ROS activity and thereby confers ROS resistance in OCCC cells. Because OCCC is exposed to free iron-induced external ROS in endometriotic cysts, as we reported previously [11], we also examined the effect of HNF1β expression under known extracellular oxidative stressors such as ferric nitrilotriacetate (FeNTA) or H_2_O_2_. Again, under such oxidative stress, control OCCC cells with high HNF1β expression had lower ROS activity levels compared with their HNF1β knockdown counterparts (Fig. 5a – 5e). These data suggest that, HNF1β expression reduces intracellular ROS activity and thereby confers ROS resistance in OCCC cells, maybe by the direct consequence of the Warburg effect.

In addition to the suppression of oxidative phosphorylation, our metabolome analysis also showed that HNF1β knockdown is associated with a significant decrease in intracellular GSH, a major regulator of intracellular ROS (Fig. 6a – 6c) [41–43]. In addition, the ratio of GSSG, an oxidized form of GSH, was also high in HNF1β knockdown cells (Fig. 6b & 6c). This suggests that active intracellular GSH is more abundant in HNF1β-high OCCC cells, which may also contribute to the ROS resistance of these cells. To explore the mechanism of the higher GSH levels in HNF1β-high OCCC cells, we measured GSS, GCLC and GCLM, the enzymes that produce GSH, but found no significant difference between HNF1β-high and low OCCC cells (Fig. 6d & 6e). Recently, several reports have shown that chemo-resistant tumor cells, including cancer stem cells, increase intracellular GSH through the cystine transporter xCT [17, 18, 44, 45]. In our experiment, cystine levels were associated with the amount of GSH in OCCC cells (Fig. 7a). However, we found no upregulation of xCT expression in HNF1β-high OCCC cells. Instead, we found that another cystine transporter, rBAT (Related to b^0,+^ Amino acid Transporter), was expressed at significantly higher levels in these cells (Fig. 6f). rBAT is coded by *rBAT* / *SLC3A1* which is well known as the responsible gene of type I cystinuria. Heterodimer of rBAT and BAT1 (b^0,+^AT), which is coded by *SLC7A9*, transports cystine and dibasic amino acids, such as lysine, arginine, and ornithine, and thus is critical for reabsorption of these amino acids in the proximal tubule. With regard to the relation of HNF1β and rBAT, Sauert K. et al. reported that HNF1β mutation decreased rBAT expression [46]. And the results of our metabolome analysis that intracellular lysine, arginine, and ornithine were significantly higher in HNF1β-high cells (Fig. S2) is compatible for high expression of rBAT in HNF1β-high OCCC cells. Furthermore, knockdown of rBAT by sh-RNA resulted in decreased levels of intracellular GSH and increased ROS activity both with and without oxidative stress (Fig. 7d – 7f), suggesting that rBAT plays a major role in HNF1β-triggered ROS resistance in OCCC cells. This is the first report to show the importance of rBAT in survival of cancer cells.

In conclusion, we have demonstrated that HNF1β drastically changes glucose and amino acid metabolism and that it promotes cell survival in hypoxic conditions under oxidative stress. These results are compatible with the fact that most OCCCs originate in endometriotic cysts and survive under severely oxidative conditions [11]. It is well known that OCCC is resistant to conventional chemotherapy and that a novel therapeutic modality is needed. Our findings here may provide several new clues to aid in the treatment of this disease (Fig. S6). First, HNF1β*-*induced aerobic glycolysis increased stress-resistance, hypoxia adaptation, and consequent survival capacity of OCCC, but these effects are highly glucose-dependent. In our assay, actually, the control RMG2 cells with higher HNF1β expression were more sensitive to glucose deprivation than were HNF1β knockdown cells, indicating that glucose uptake and metabolism may be a therapeutic target in OCCC. Secondly, HNF1β inhibition with some type of inhibitor, such as the microRNA mir-802 [51], may have a therapeutic effect by abrogating ROS resistance in OCCC. Finally, an inhibitor of GSH synthesis, such as buthionine sulfoximine [52, 53], or inhibition of GSH synthesis by targeting the cystine transporter rBAT, may enhance the effect of conventional chemotherapy. Further study is needed to explore the potential of these therapeutic applications.

## MATERIALS AND METHODS

### Cell lines and cell culture

The OCCC cell line RMG2 was kindly provided by Dr. Aoki at Keio University, and the OCCC cell line JHOC5 was purchased from the Riken BioResource Center. Cells were cultured in RPMI1640 medium (Nacalai Tesque, Kyoto, Japan) supplemented with 10% fetal bovine serum (FBS) (Biowest, Courtaboeuf Cedex, France) and penicillin–streptomycin (Nacalai Tesque) in a humidified atmosphere containing 5% CO_2_ at 37°C.

RMG2 and JHOC5 knockdown cells were generated by lentiviral transfection using two different *GIPZ* Lentiviral shRNAs and a nonsilencing control RNA (Thermo Scientific, Waltham, MA) (cone name/ sh RNA clone ID/ antisense sequence: *HNF1β*_ sh1/ V2LHS_204881/ TGAATTGTCGGAGGA TCTC, *HNF1β*_sh2/ V3LHS_409658/ AGTTTATAG TTTACAGCCA, *rBAT*- sh1/ V2LHS_229777/ TATGTT TATCACTCGTGTG, *rBAT*- sh2/ V3LHS_311025/ ATTG TGTGACCGTGTCCGG, control/ RHS4348). We used 10 μg /ml puromycin for selection and confirmed that over 95% of cells were successfully transfected with RNA based on GFP fluorescence by fluorescence microscopy. We checked the transfection efficacies of *HNF1β* and *rBAT* by RT-PCR and western blotting (Fig. S4a, S4b, S4c, & S4d).

### Real-time polymerase chain reaction (RT-PCR)

Total RNA was isolated using the RNeasy Mini Kit (Qiagen, Tokyo, Japan), treated with the PrimeScript™ RT reagent Kit (Takara, Shiga, Japan), and analyzed using Light Cycler 480 Real-Time PCR system (Roche, Basel, Switzerland). The *HNF1β*, *rBAT* and *HPRT1* (housekeeping gene) primers and probes were designed using Roche Universal ProbeLibrary Assay Design Center software (http://qpcr.probefinder.com/roche3.html) (gene name/ assession number/ antisense primer sequence/ sense primer sequence/ Roche Universal Probe # : *HNF1β*/ NM_000458.2/ caccaacatgtcttcaagtaaacag/ ttgttgcgcacgaagtaagt/ 9, *rBAT*/ NM_000341.3/ accacactgtg aatgttgatgtc/ agtagctcattggcatgaagtaga/ 26, *HPRT1*/ NM_000194.2/ tgaccttgatttattttgcatacc/ cgagcaagacgttc agtcct/ 73).

### Intracellular ROS analysis

Each cell culture, both with and without oxidative stress, was washed twice with PBS and then stained with 5 μM CellROX^®^ Deep Red Reagent (Life Technologies, Japan, Tokyo) with complete medium at 37°C for 30 minutes. The cells were then harvested and washed with PBS and analyzed using a FACSCalibur cytometer (Becton Dickinson, Franklin Lakes, NJ). The results were analyzed using FlowJo_V10 software. Differences between groups were assessed based on the mean fluorescence intensity (MFI) of FL4.

### Intracellular GSH and GSSG analysis

GSH was measured using a GSH/GSSG quantification assay kit (Dojindo, Kumamoto, Japan), based on the enzymatic recycling method. Cultured cells were harvested, treated, measured by absorption spectrometer, and quantified according to the manufacturer's manual. (http://www.dojindo.com/store/p/824-GSSG-GSH-Quantification-Kit.html).

### Comprehensive analysis of intracellular metabolites

RMG2_*HNF1β*_sh1 cells and RMG2_control cells cultured with complete medium (D-glucose 2000 mg / l, L-glutamine 300 mg / l, glutathione 1 mg / l, lactate 0 mg / l, and pyruvate 0 mg / l) at 37°C in 20% O2 and 5% CO_2_ were washed by mannitol solution, quenched by methanol and collected. Then, 5 × 10^6^ cells from each condition were diluted with 1000 μl chloroform and 400 μl distilled water, and then centrifuged at 2300 × g for 5 minutes at 4°C. Replicate 400 μl aliquots of each supernatant (for anion/cation) were centrifuged at 9100 × g for 120 minutes at 4°C using a 5 kDa ultrafiltration membrane (Millipore, Billerica, MA). The supernatant from each sample was solidified, re-diluted with 50 μl distilled water, and measured by CE-TOFMS (Capillary electrophoresis electrospray ionization time-of-flight mass spectrometry). This measurement was performed by Human Metabolome Technologies (HMT) Inc. (Yamagata, Japan). Based on HMT's database of the mass-to-charge ratio (m/z) and the migration time (MT) of peaks detected in CE-TOFMS, 193 peaks were assigned to candidate metabolites. Of these 193 metabolites, 87 were quantified using integrated peak areas obtained from measurements of chemical standards that were analyzed in parallel with experimental samples. Others were compared using their relative peak areas. Data were analyzed using previously reported methods [54, 55].

### Microarray analysis

GSE39204 [56], which contained clinical ovarian cancer specimens from 64 patients (16 OCCC and 48 non-OCCC (29 serous, 13 endometrioid, 2 mucinous, and 4 others)) who underwent primary surgery at Kyoto University Hospital between 1997 and 2011, was used for GSEA and analysis of metabolic-related gene expression.

GSE6008, which was uploaded to publicly available online databases by Wu R et al. and contained 99 individual ovarian tumors (8 OCCC and 91 non-OCCC (41 serous, 37 endometrioid, 13 mucinous, and 4 individual normal ovary samples)) [30], was also used for GSEA.

GSE37290 [25], a microarray dataset of RMG2 *HNF1β*_sh1 cells and RMG2 control cells, was also analyzed to evaluate the relevance of *HNF1β* to metabolic function in OCCC.

Total RNA expression was analyzed on the HG U133 Plus 2.0 Array (Affymetrix) (GSE39204 and GSE37290) and the HG_U133A array (Affymetrix) (GSE6008). The expression intensities were normalized across all samples using the robust multichip average algorithm [57]. Gene set enrichment analysis (GSEA) was conducted using GSEA software (http://www.broadinstitute.org/gsea/downloads.jsp).

### Western blot analysis

Whole cell lysates were prepared for western blot analysis. Equal amounts of protein were electrophoresed on SDS–PAGE gels and transferred to polyvinylidene fluoride membranes (Bio-Rad). Then, they were probed with the following antibodies: HNF1β (sc-7411, 1:200; Santa Cruz Biotechnology, Dallas, TX); rBAT/SLC3A1 (ab104691, 1:200, Abcam); β-Actin (ab8227, 1:3000, Abcam), and visualized by Chemi-Lumi One (Nacalai Tesque) and the ChemiDoc™ XRS+ system (Bio-Rad, Hercules, CA).

### Oxidative stress / CDDP resistance analysis

Oxidative stress resistance was assessed by MTT assay using WST-8 (Cell Count Reagent SF, Nacalai Tesque) after treatment with oxidative agents (ferric nitrilotriacetate or hydrogen peroxide) or CDDP. The concentration of oxidative agents required to inhibit cell proliferation by 50% (IC50) was calculated using Prism 5 software (GraphPad, California, USA).

### Cell proliferation under hypoxia

Cells were pre-cultured in 20% O_2_ for 24 hours and then in either 2% O_2_ (hypoxia) or 20% O_2_ (normoxia). Cell proliferation under hypoxia and normoxia was assessed by MTT assay using WST-8 (Cell Count Reagent SF) every 24 hours.

### Glucose deprivation assay

Cells were pre-cultured in complete medium (D-glucose 2000 mg / l, L-glutamine 300 mg / l, glutathione 1 mg / l, lactate 0 mg / l, and pyruvate 0 mg / l) at 37°C in 20% O2 and 5% CO_2_ for 72 hours as previously described and then cultured in either glucose (+) medium (D-glucose 2000 mg / l, L-glutamine 300 mg / l, glutathione 1 mg / l, lactate 0 mg / l, and pyruvate 0 mg / l, Nacalai RPMI1640 cat. no. 30264), or glucose (−) medium (D-glucose 0 mg / l, L-glutamine 300 mg / l, glutathione 1 mg / l, lactate 0 mg / l, and pyruvate 0 mg / l, Nacalai RPMI1640 cat. no. 09892). Both media were supplemented with 10% FBS and contained the same formulation except for the presence or absence of D-glucose. Cell proliferation was assessed by MTT assay using WST-8 (Cell Count Reagent SF) every 24 hours.

### Statistical analyses

Statistical significance was assessed by two-tailed unpaired *t*-tests using Prism 5 software (GraphPad), and *p* values of < 0.05 were considered statistically significant. Data are shown as the mean ± standard deviation (SD).

## SUPPLEMENTARY FIGURES AND TABLES



## Abbreviations

HNF1β: hepatocyte nuclear factor 1β
OCCC: ovarian clear cell carcinoma
ROS: reactive oxygen species
GSH: glutathione
GSEA: gene set enrichment analysis
FeNTA: ferric nitrilotriacetate
PPP: pentose-phosphate-pathway
