# Supplementary material for: Metabolic alterations caused by HNF1β expression in ovarian clear cell carcinoma contribute to cell survival
Source: Oncotarget. 2015 Jul 31;6(28):26002–17. doi: 10.18632/oncotarget.4692 (PMC4694881; doi:10.18632/oncotarget.4692)
Supplement: Supplementary file 1 [file oncotarget-06-26002-s001.pdf]

SUPPLEMENTARY FIGURES AND TABLES

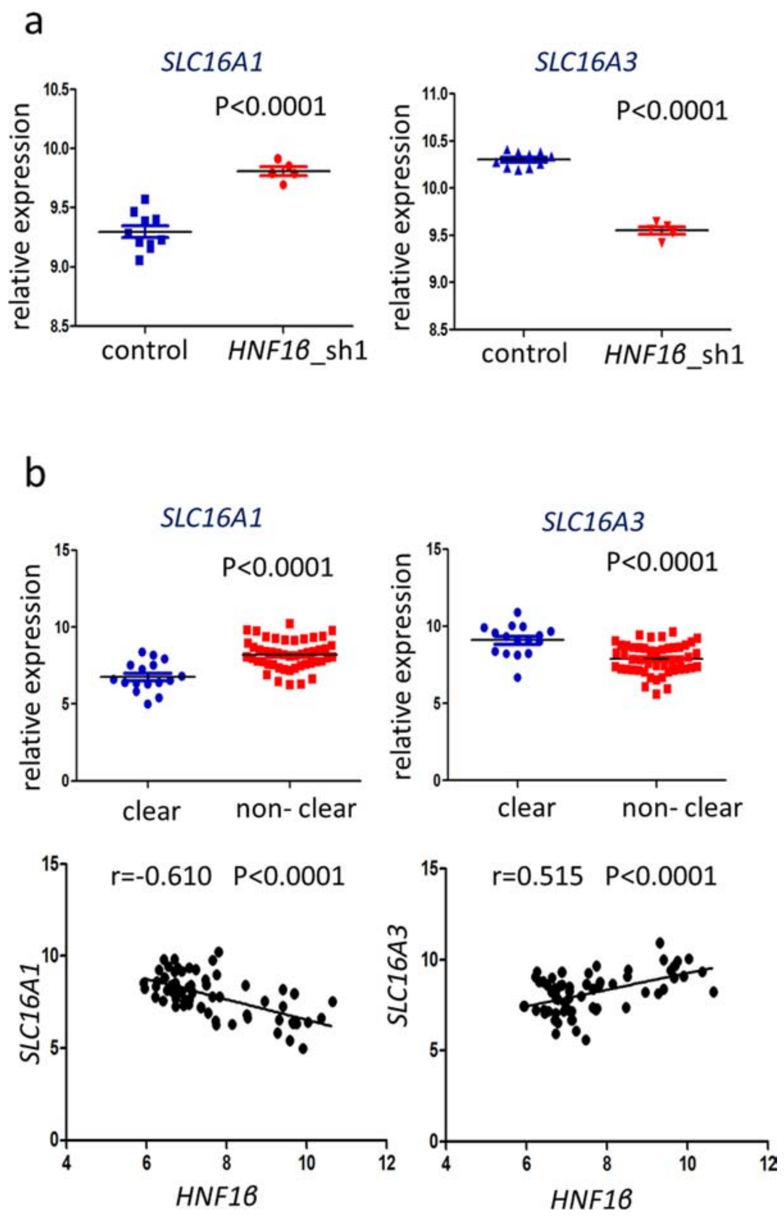

Supplementary Figure S1: HNF1β expression had a negative correlation to the lactic acid import transporter MCT1, and a positive correlation to the lactic acid export transporter MCT4. a, b. The correlations of *HNF1β* expression and expression of *SLC16A1* that codes lactic acid import transporter MCT1, and expression of *SLC16A3* that codes lactic acid export transporter MCT4 were analyzed by microarray data of (a) RMG2 *HNF1β* knockdown model dataset GSE37290 and (b) the clinical dataset GSE39204.

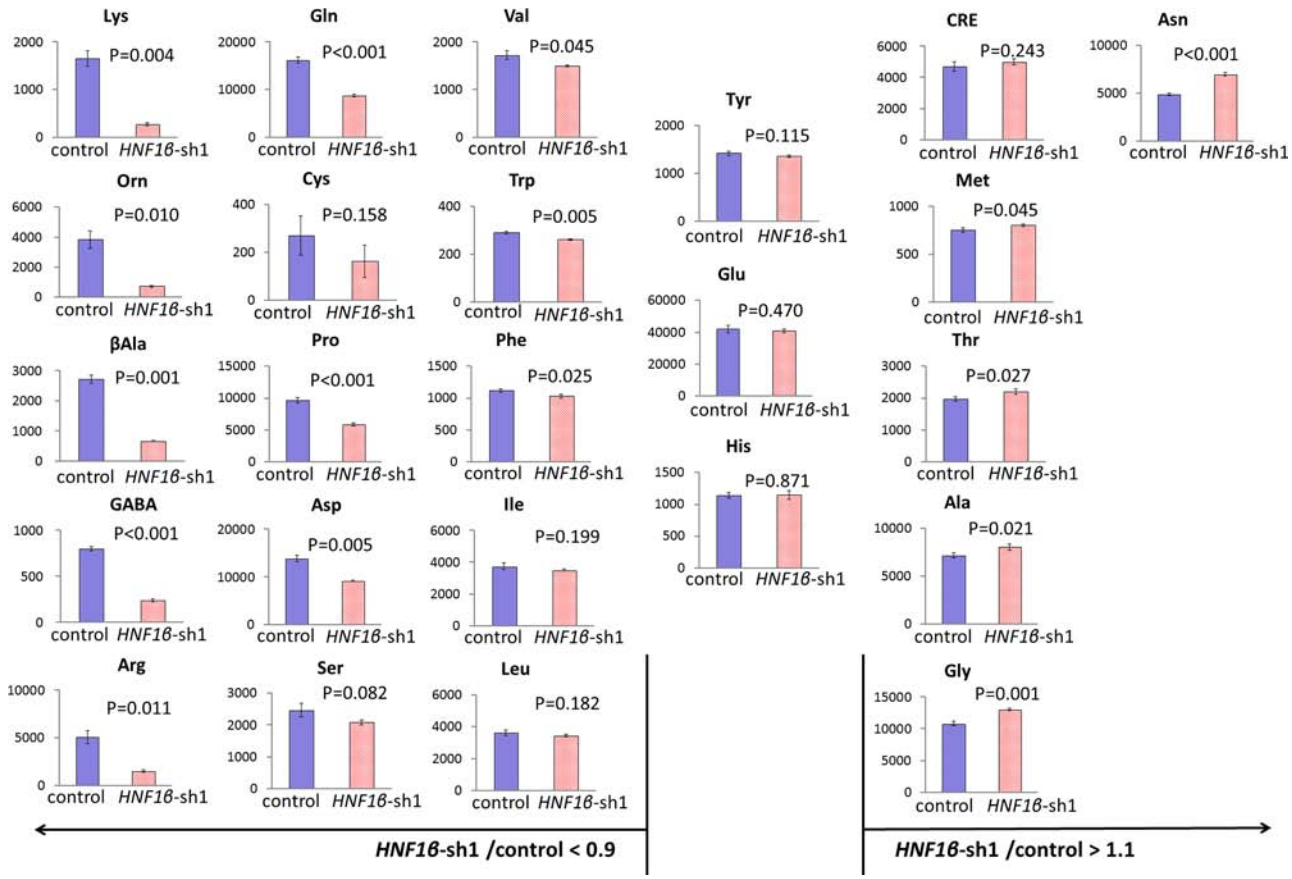

Supplementary Figure S2: HNF1β knockdown dynamically changed intracellular amino acids. In the metabolome analysis of RMG2, of the 20 amino acids, 13 were significantly altered in *HNF1β*-sh1 cells. Cysteine, lysine, arginine, glutamate, proline, and aspartate were dynamically decreased, but asparagine, glycine, and alanine were dynamically increased by *HNF1β* knockdown. The amount of substrate used was pmol /10^6 cells. $n = 3$.

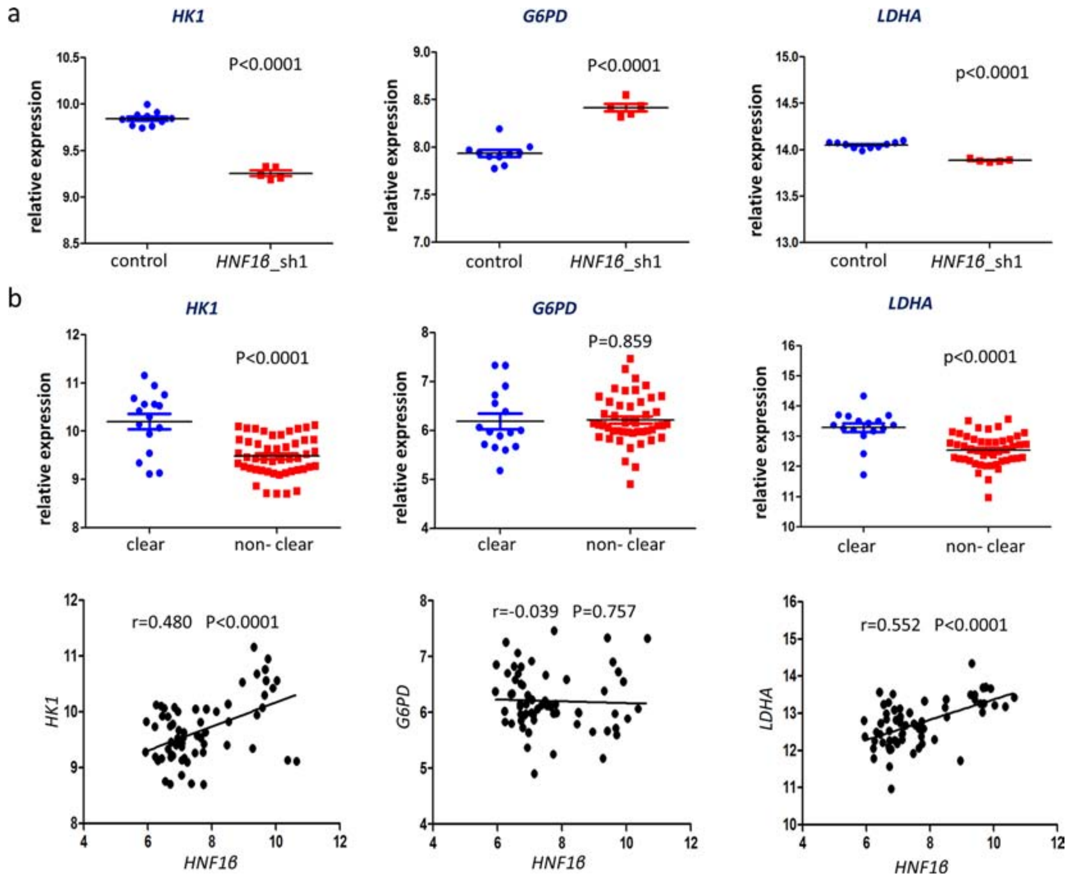

Supplementary Figure S3: HNF1β expression had a positive correlation not to G6PD but to HK1 and LDHA that were known as key genes of aerobic glycolysis. a, b. Expression of key genes of aerobic glycolysis (*HK1*, *G6PD*, and *LDHA*) based on (a) RMG2 microarray datasets GSE37290 and (b) the clinical dataset GSE39204.

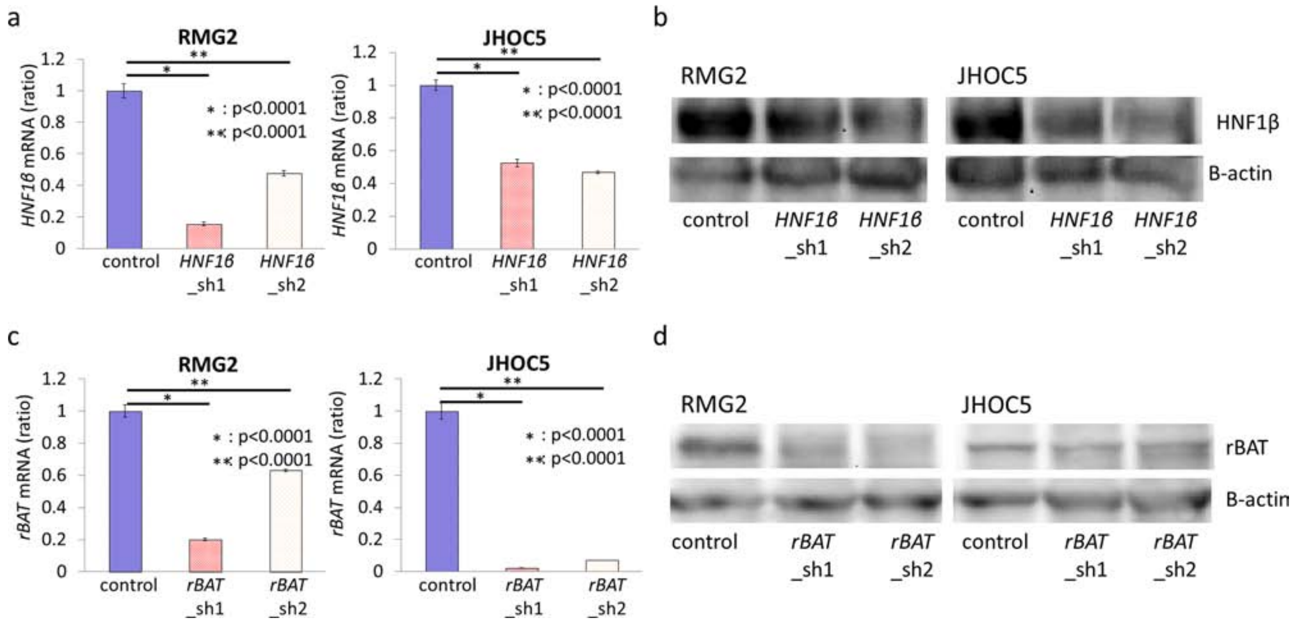

Supplementary Figure S4: Efficiency of knockdown in stable HNF1β and rBAT knockdown cells using shRNA transfection. a. Stable HNF1β knockdown were established by transfection with 2 types of short hairpin RNA or control RNA into RMG2 and JHOC5 cells. mRNA expression of *HNF1β* detected by qRT-PCR was effectively decreased by stable knockdown both in RMG2 and JHOC5. $n = 4$. **b.** A total of 30 μg whole cell lysates of HNF1β knockdown RMG2 and JHOC5 cells were electrophoresed on SDS–PAGE gels, transferred to polyvinylidene fluoride membranes and probed with the following antibodies: HNF1β (1:200); β-Actin (1:3000). Protein levels of HNF1β were also decreased in *HNF1β*_sh RMG2 and JHOC5 cells. **c.** Stable rBAT knockdown were established by transfection with 2 types of short hairpin RNA or control RNA into RMG2 and JHOC5 cells. *rBAT* mRNA expression was effectively decreased by stable knockdown both in RMG2 and JHOC5. $n = 4$. **d.** A total of 30 μg whole cell lysates of rBAT knockdown RMG2 and JHOC5 cells were electrophoresed on SDS–PAGE gels, transferred to polyvinylidene fluoride membranes and probed with the following antibodies: rBAT (1:200); β-Actin (1:3000). Protein levels of rBAT were also decreased in *rBAT*_sh RMG2 and JHOC5 cells.

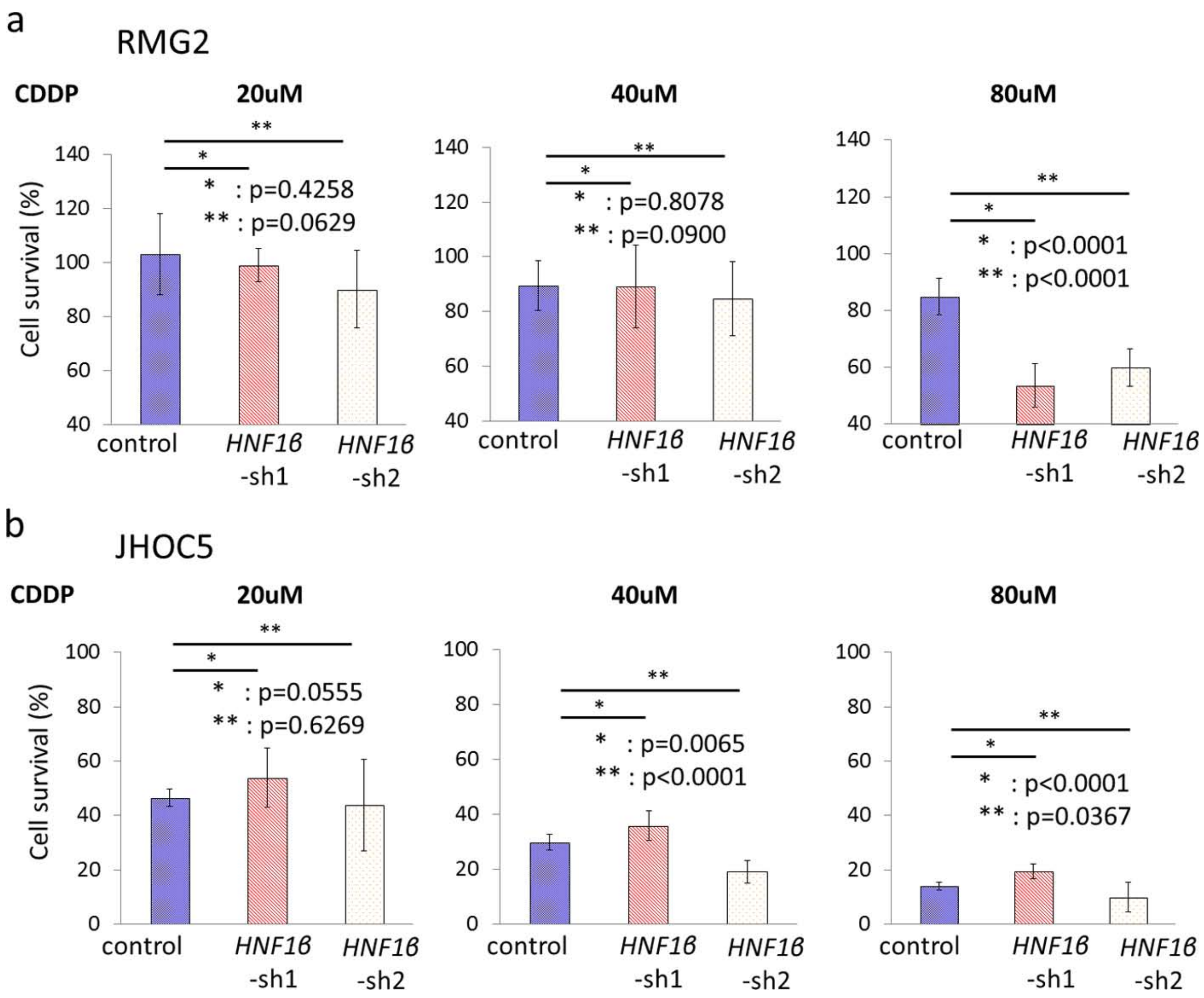

Supplementary Figure S5: CDDP resistance of RMG2 and JHOC5 were not significantly altered by HNF1β knockdown under normoxia. a, b. Control, *HNF1β*_sh1, and *HNF1β*_sh2 cells were exposed to 0, 20, 40, or 80 µM CDDP for 24 hours in 20% O_2. Cell survival was calculated as the WST-8 value of CDDP 20, 40, or 80 µM divided by those in the absence of CDDP (CDDP 0 µM) in (a) RMG2 and (b) JHOC5 cells. *n* = 10.

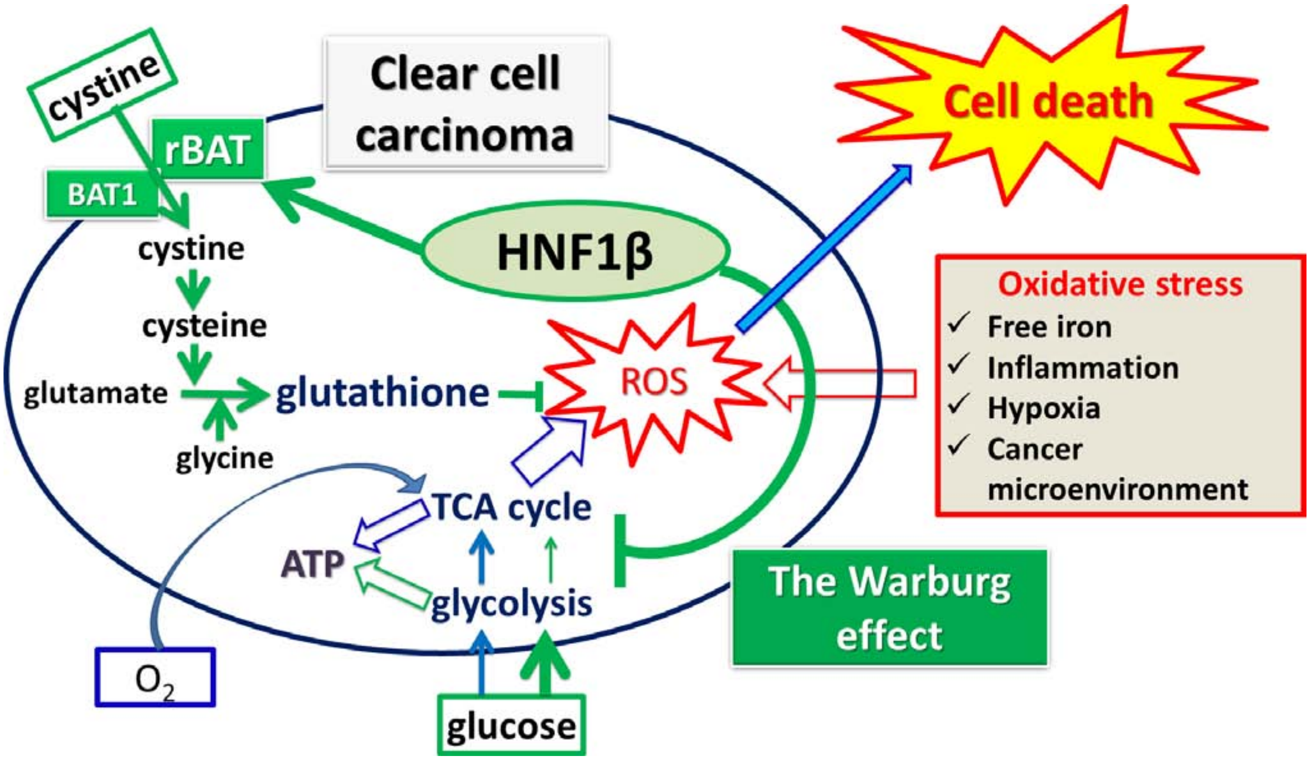

Supplementary Figure S6: Schema of HNF1β induced metabolic alteration in OCCC cells. Green (but not blue) color in the schema represents metabolic processes caused by HNF1β. First, HNF1β-induced aerobic glycolysis increases stress-resistance and survival capacity of OCCC in exchange for glucose-dependency. Second, HNF1β enhances ROS resistance via GSH synthesis through cystine transporter rBAT expression. These mechanisms are supposed to be the potential targets of novel therapeutic applications.

Supplementary Table S1: a, b. Go terms enriched in the OCCC group, based on the GSEA analysis of (a) the GSE39204 dataset and (b) the GSE6008 dataset. Gene sets written in ***bold letters*** are related to metabolism, and those in ***italic letters*** are common in both GSE39204 and GSE6008. **c.** Transcription motifs enriched in the OCCC group, based on the GSEA analysis of each the GSE39204 and the GSE6008 dataset. Transcription motifs that are common in both datasets are written in **bold letters**.

a. Enriched Go terms in OCCC_GSE39204

Go term NAME	*NES	*p*-value
NUCLEOTIDE_METABOLIC_PROCESS	1.743445	0.004000
FATTY_ACID_OXIDATION	1.721238	0.008197
PROTEIN_AMINO_ACID_LIPIDATION	1.718183	0.019841
PROTEIN_HOMOOLIGOMERIZATION	1.706216	0.002058
INTERACTION_WITH_HOST	1.681972	0.006438
PERINUCLEAR_REGION_OF_CYTOPLASM	1.660041	0.013645
RECEPTOR_SIGNALING_PROTEIN_SERINE_THREONINE_KINASE_ACTIVITY	1.659409	0.010060
NUCLEOBASENUCLEOSIDE_AND_NUCLEOTIDE_METABOLIC_PROCESS	1.654287	0.005929
GOLGI_APPARATUS_PART	1.647091	0.016327
GOLGI_APPARATUS	1.64647	0.018634
AMINO_ACID_METABOLIC_PROCESS	1.640557	<0.000001
PEROXISOME_ORGANIZATION_AND_BIOGENESIS	1.625024	0.019194
AMINO_ACID_AND_DERIVATIVE_METABOLIC_PROCESS	1.615956	0.002062
ORGANIC_ACID_METABOLIC_PROCESS	1.579711	0.004425
RESPONSE_TO_OXIDATIVE_STRESS	1.575047	0.010823
SECONDARY_METABOLIC_PROCESS	1.572381	0.018182
**CELLULAR_CATABOLIC_PROCESS**	1.571581	0.008421
CARBOXYLIC_ACID_METABOLIC_PROCESS	1.565290	0.004435
*TRANSFERASE_ACTIVITY_TRANSFERRING_ACYL_GROUP*S	1.559770	0.021053
OXIDOREDUCTASE_ACTIVITY_ACTING_ON_THE_CH_CH_GROUP_OF_DONORS	1.545005	0.028571
AMINE_METABOLIC_PROCESS	1.542132	0.008658
NITROGEN_COMPOUND_METABOLIC_PROCESS	1.539846	0.006550
TRANS_GOLGI_NETWORK	1.536914	0.024896
**OXYGEN_AND_REACTIVE_OXYGEN_SPECIES_METABOLIC_PROCESS**	1.534516	0.030738
ORGANIC_ACID_TRANSPORT	1.505359	0.027254
SODIUM_ION_TRANSPORT	1.499128	0.040650
POSITIVE_REGULATION_OF_TRANSCRIPTION_FROM_RNA_POLYMERASE_II_PROMOTER	1.497856	0.047337
SH3_SH2_ADAPTOR_ACTIVITY	1.49246	0.030829

(*Continued*)

a. Enriched Go terms in OCCC_GSE39204

Go term NAME	*NES	*p*-value
**CATABOLIC_PROCESS**	1.491078	0.018828
STRESS_ACTIVATED_PROTEIN_KINASE_SIGNALING_PATHWAY	1.489182	0.022680
ATPASE_ACTIVITY_COUPLED	1.483661	0.024096
**STEROID_METABOLIC_PROCESS**	1.480793	0.045455
JNK_CASCADE	1.472951	0.030992
**CELLULAR_LIPID_METABOLIC_PROCESS**	1.462200	0.033403
PROTEIN_COMPLEX_ASSEMBLY	1.456123	0.004246
MOLECULAR_ADAPTOR_ACTIVITY	1.446257	0.034615
ISOMERASE_ACTIVITY	1.434808	0.03675
ATP_BINDING	1.430988	0.014737
ORGANIC_ACID_TRANSMEMBRANE_TRANSPORTER_ACTIVITY	1.429714	0.031983
PROTEIN_OLIGOMERIZATION	1.428117	0.042373
ADENYL_RIBONUCLEOTIDE_BINDING	1.422954	0.024948
ATPASE_ACTIVITY_COUPLED_TO_TRANSMEMBRANE_ MOVEMENT_OF_IONS_PHOSPHORYLATIVE_MECHANISM	1.421074	0.049080
**LIPID_METABOLIC_PROCESS**	1.403919	0.040984
OXIDOREDUCTASE_ACTIVITY	1.400326	0.023041
ADENYL_NUCLEOTIDE_BINDING	1.370103	0.034765
PURINE_RIBONUCLEOTIDE_BINDING	1.310640	0.043388

b. Enriched Go terms in OCCC_GSE6008

Go term NAME	*NES	*p*-value
INTERACTION_WITH_HOST	1.899776	<0.000001
POSITIVE_REGULATION_OF_TRANSLATION	1.897164	<0.000001
TRANS_GOLGI_NETWORK	1.768277	0.020040
NUCLEOBASENUCLEOSIDE_AND_NUCLEOTIDE_METABOLIC_ PROCESS	1.767408	0.002101
NUCLEOTIDE_METABOLIC_PROCESS	1.752282	0.004292
RECEPTOR_SIGNALING_PROTEIN_SERINE_THREONINE_KINASE_ ACTIVITY	1.731334	0.015779
SH3_SH2_ADAPTOR_ACTIVITY	1.726792	<0.000001
HYDROLASE_ACTIVITY_ACTING_ON_CARBON_NITROGEN_ NOT_PEPTIDEBONDSIN_CYCLIC_AMIDINES	1.723641	0.004175
POSITIVE_REGULATION_OF_CASPASE_ACTIVITY	1.704000	0.003899
**HETEROCYCLE_METABOLIC_PROCESS**	1.695265	0.015086
**PIGMENT_METABOLIC_PROCESS**	1.688258	0.008282
PROTEIN_BINDING_BRIDGING	1.683446	0.006356
REGULATION_OF_MAPKKK_CASCADE	1.641133	0.012000

(*Continued*)

b. Enriched Go terms in OCCC_GSE6008		
**Go term NAME**	***NES**	***p*-value**
CASPASE_ACTIVATION	1.612209	0.014056
MOLECULAR_ADAPTOR_ACTIVITY	1.592820	0.006148
L_AMINO_ACID_TRANSMEMBRANE_TRANSPORTER_ ACTIVITY	1.590038	0.010941
AMINO_ACID_METABOLIC_PROCESS	1.590001	0.010526
PERINUCLEAR_REGION_OF_CYTOPLASM	1.586397	0.021231
AMINO_ACID_TRANSMEMBRANE_TRANSPORTER_ACTIVITY	1.584290	0.012605
STRESS_ACTIVATED_PROTEIN_KINASE_SIGNALING_PATHWAY	1.580877	0.015779
RESPONSE_TO_NUTRIENT	1.580422	0.010183
POSITIVE_REGULATION_OF_TRANSCRIPTION_FROM_RNA_ POLYMERASE_II_PROMOTER	1.578651	0.009862
**CYTOKINE_METABOLIC_PROCESS**	1.574696	0.012903
PIGMENT_BIOSYNTHETIC_PROCESS	1.569508	0.033333
TRANSFERASE_ACTIVITY_TRANSFERRING_ACYL_GROUPS	1.561541	0.007968
G_PROTEIN_SIGNALING_ADENYLATE_CYCLASE_ ACTIVATING_PATHWAY	1.544681	0.026369
RESPONSE_TO_HORMONE_STIMULUS	1.544023	0.030238
CYTOKINE_BIOSYNTHETIC_PROCESS	1.541688	0.019149
POSITIVE_REGULATION_OF_CYTOKINE_BIOSYNTHETIC_ PROCESS	1.539349	0.042222
REGULATION_OF_CYTOKINE_BIOSYNTHETIC_PROCESS	1.538080	0.023305
AMINE_TRANSMEMBRANE_TRANSPORTER_ACTIVITY	1.536809	0.010309
VACUOLE	1.536172	0.021552
RESPONSE_TO_HYPOXIA	1.534777	0.039920
JNK_CASCADE	1.530886	0.019802
AMINO_ACID_TRANSPORT	1.529789	0.021739
T_CELL_PROLIFERATION	1.52085	0.035011
EXOCYTOSIS	1.518632	0.023758
PROTEIN_HOMOOLIGOMERIZATION	1.51606	0.032051
REGULATION_OF_TRANSLATION	1.510512	0.010776
ACTIVE_TRANSMEMBRANE_TRANSPORTER_ACTIVITY	1.507351	0.006522
ORGANIC_ACID_TRANSMEMBRANE_TRANSPORTER_ACTIVITY	1.501320	0.017391
ORGANIC_ACID_TRANSPORT	1.495730	0.016949
ORGANIC_ACID_METABOLIC_PROCESS	1.491943	0.021142
AMINE_METABOLIC_PROCESS	1.490689	0.010549
HYDROLASE_ACTIVITY_HYDROLYZING_O_GLYCOSYL_ COMPOUNDS	1.488564	0.042373
RESPONSE_TO_EXTRACELLULAR_STIMULUS	1.485037	0.031579

(*Continued*)

b. Enriched Go terms in OCCC_GSE6008		
**Go term NAME**	***NES**	***p*-value**
SECONDARY_METABOLIC_PROCESS	1.483332	0.040426
EXTRACELLULAR_MATRIX_STRUCTURAL_CONSTITUENT	1.480458	0.026971
HYDROLASE_ACTIVITY_ACTING_ON_CARBON_NITROGEN_NOT_PEPTIDEBONDS	1.473416	0.045267
CARBOXYLIC_ACID_METABOLIC_PROCESS	1.468384	0.021368
NITROGEN_COMPOUND_METABOLIC_PROCESS	1.466930	0.008658
TUBULIN_BINDING	1.464725	0.047337
ATPASE_ACTIVITY_COUPLED	1.462013	0.028889
HOMEOSTASIS_OF_NUMBER_OF_CELLS	1.458903	0.042945
GOLGI_APPARATUS	1.443387	0.016913
PROTEIN_OLIGOMERIZATION	1.442648	0.041176
AMINO_ACID_AND_DERIVATIVE_METABOLIC_PROCESS	1.438608	0.023355
AMINE_TRANSPORT	1.431743	0.039841
TRANSLATION	1.429832	0.034783
HYDROLASE_ACTIVITY_ACTING_ON_ACID_ANHYDRIDESCATALYZING_TRANSMEMBRANE_MOVEMENT_OF_SUBSTANCES	1.424451	0.030837
ATPASE_ACTIVITY_COUPLED_TO_MOVEMENT_OF_SUBSTANCES	1.417552	0.031320
PRIMARY_ACTIVE_TRANSMEMBRANE_TRANSPORTER_ACTIVITY	1.416358	0.033482
MACROMOLECULE_BIOSYNTHETIC_PROCESS	1.384657	0.037296
TRANSCRIPTION_COACTIVATOR_ACTIVITY	1.338881	0.045545
CELL_DEVELOPMENT	1.307402	0.026432
TRANSCRIPTION_ACTIVATOR_ACTIVITY	1.307166	0.040000
CELLULAR_BIOSYNTHETIC_PROCESS	1.299733	0.038627
CELL_FRACTION	1.272983	0.043678
SENSORY_PERCEPTION	1.272934	0.046025
**POSITIVE_REGULATION_OF_METABOLIC_PROCESS**	1.271422	0.050000
PHOSPHOTRANSFERASE_ACTIVITY_ALCOHOL_GROUP_AS_ACCEPTOR	1.261705	0.043299
NEUROLOGICAL_SYSTEM_PROCESS	1.235814	0.035477

c. Enriched transcription motifs in OCCC		
**GSE39204**		
**NAME**	***NES**	***p*-value**
**RGTTAMWNATT_V$HNF1_01**	1.6800531	0.0020450
V$PPARG_01	1.6574575	0.0244898
V$PPARA_01	1.4561901	0.0498915

(*Continued*)

c. Enriched transcription motifs in OCCC

GSE39204

NAME	*NES	*p*-value
V$HNF4_DR1_Q3	1.4259486	0.0165631
**V$SF1_Q6**	1.4047260	0.0242915
V$HNF4_01_B	1.3439399	0.0351648
V$COUP_01	1.3162062	0.0416667
V$DR1_Q3	1.2827437	0.0443160

GSE6008

NAME	*NES	*p*-value
YTCCCRNNAGGY_UNKNOWN	1.7393075	0.0083507
**RGTTAMWNATT_V$HNF1_01**	1.6671798	0.0061350
V$NFE2_01	1.4761780	0.0170576
**TGACCTTG_V$SF1_Q6**	1.4166850	0.0182232
AAGWWRNYGGC_UNKNOWN	1.4048319	0.0241228
CATRRAGC_UNKNOWN	1.4040798	0.0482180
V$HNF4ALPHA_Q6	1.3904574	0.0482180
V$MYOD_Q6	1.3475660	0.0210526
V$USF_01	1.3301750	0.0432990
V$NFAT_Q4_01	1.2987286	0.0491453
V$DR3_Q4	1.2929122	0.0442478

*NES : normalized enrichment score

Supplementary Table S2: a. Intracellular metabolites that decreased by at least 0.7-fold in HNF1β knockdown cells. **b.** Intracellular metabolites that increased by at least 1.4-fold in HNF1β knockdown cells.

**a. metabolites decreased by at least 0.7-fold in HNF1B_sh1 cells**		
**Compound name**	**HNF1B_sh1 / control ratio**	***p*-value**
Phytic acid_divalent	< 0.001	N.A.
Ethanolamine phosphate	0.0924933	0.023944
Argininosuccinic acid	0.1437473	0.001092
Lys	0.1655898	0.004085
dUMP	0.1843451	0.001483
Streptomycin sulfate_+H2O_divalent	0.1847339	0.465400
Ornithine	0.1908658	0.010274
Taurine	0.2197816	0.000510
Hypotaurine	0.2243896	0.000652
Carnitine	0.2312600	0.002788
5-Aminoimidazole-4-carboxamide ribotide	0.2415160	0.004709
β-Ala	0.2435182	0.001037
CDP-choline	0.2466622	0.016057
GABA	0.2986792	6.73E-06
Arg	0.3014228	0.010578
Carnosine	0.3261855	0.003447
Gluconic acid	0.3310880	0.004750
Spermine	0.3756026	0.192670
SDMA	0.3801309	0.001618
Cystathionine	0.3835219	1.83E-07
N6-Methyllysine	0.3840033	0.022116
ADMA	0.4013655	6.44E-05
Glucuronic acid	0.4184396	0.016984
N-Acetylneuraminic acid	0.4187018	3.86E-06
O-Acetylcarnitine	0.4272483	0.001221
3-Aminoisobutyric acid	0.4286996	0.000396
γ-Butyrobetaine	0.4301724	9.25E-05
2-Deoxyglucose 6-phosphate	0.4366955	0.138840
1-Methylnicotinamide	0.4572230	0.004518
p-Aminobenzoic acid	0.4754198	0.000331
dTMP	0.4874561	0.061231
Isethionic acid	0.5184781	0.004865
Thiamine	0.5241244	0.026725
Gln	0.5395338	0.000591

(*Continued*)

a. metabolites decreased by at least 0.7-fold in HNF1B_sh1 cells

Compound name	HNF1B_sh1 / control ratio	*p*-value
Betaine	0.5598650	0.002018
5-Aminovaleric acid	0.5890031	0.012546
Cys	0.6043437	0.158180
Pro	0.6086360	0.000792
Pelargonic acid	0.6094951	0.031894
Glutathione (GSH)	0.6281293	0.000292
o-Aminophenol	0.6318320	0.050620
Octanoic acid	0.6389915	0.020638
Putrescine	0.6402218	0.569890
Urea	0.6460634	0.003254
Choline	0.6468962	0.019406
Asp	0.6627155	0.005443
Malic acid	0.6715178	0.004466
m-Toluic acid	0.6777080	0.289230
1-Pyrroline 5-carboxylic acid	0.6815753	0.047168
Heptanoic acid	0.6902770	0.215110
γ-Glu-Cys	0.6910592	0.003776
GMP	0.6994325	0.010826

b. metabolites increased by at least 1.4-fold in HNF1B_sh1 cells

Compound name	HNF1B_sh1 / control ratio	*p*-value
Diethanolamine	>1000	N.A.
Hippuric acid	7.448096080	0.003009
myo-Inositol 1-phosphate	3.585767801	0.008525
myo-Inositol 3-phosphate		
3-Hydroxy-3-methylglutaric acid	2.681647422	0.012076
N8-Acetylspermidine	2.147171885	0.110500
Ribulose 1, 5-diphosphate	2.145266277	0.013997
Glycerophosphocholine	2.067160815	0.000301
Glutaric acid	2.053786618	0.000161
trans-Glutaconic acid	1.777829295	0.023911
Uric acid	1.629715316	0.000127
Adenosine 5′-phosphosulfate	1.627766572	0.001434
2-Amino-2-(hydroxymethyl)-1, 3-propanediol	1.622278425	0.502130
6-Aminohexanoic acid	1.607716008	0.021992
Ribulose 5-phosphate	1.538751060	0.010237

(*Continued*)

b. metabolites increased by at least 1.4-fold in HNF1B_sh1 cells		
**Compound name**	**HNF1B_sh1 / control ratio**	***p*-value**
Pyruvic acid	1.478190263	0.005594
2-Oxoglutaric acid	1.455141269	0.001230
N-Acetyl-β-alanine	1.445945287	0.000759
Pyridoxal	1.433917277	0.132540
Asn	1.428807590	0.000558
